# Cerebral lateralisation of first and second languages in bilinguals assessed using functional transcranial Doppler ultrasound

**DOI:** 10.12688/wellcomeopenres.9869.1

**Published:** 2016-11-15

**Authors:** Clara R. Grabitz, Kate E. Watkins, Dorothy V. M. Bishop

**Affiliations:** 1 Department of Experimental Psychology, University of Oxford, Oxford, UK

**Keywords:** Laterality, Bilingualism, FTCD

## Abstract

**Background:** Lateralised representation of language in monolinguals is a well-established finding, but the situation is much less clear when there is more than one language. Studies to date have identified a number of factors that might influence the brain organisation of language in bilinguals. These include proficiency, age of acquisition and exposure to the second language. The question as to whether the cerebral lateralisation of first and second languages are the same or different is as yet unresolved.

**Methods:** We used functional transcranial Doppler sonography (FTCD) to measure cerebral lateralisation in the first and second languages in 26 high proficiency bilinguals with German or French as their first language (L1) and English as their second language (L2). FTCD was used to measure task-dependent blood flow velocity changes in the left and right middle cerebral arteries during word generation cued by single letters. Language history measures and handedness were assessed through self-report questionnaires.

**Results:**The majority of participants were significantly left lateralised for both L1 and L2, with no significant difference in the size of asymmetry indices between L1 and L2. Asymmetry indices for L1 and L2 were not related to language history, such as proficiency of the L2.

**Conclusion:*** *In highly proficient bilinguals, there is strong concordance for cerebral lateralisation of first and second languages.

## Introduction

The two cerebral hemispheres of the brain are neither structurally nor functionally identical. Hemispheric specialisation reflects a variety of factors influencing brain development, including genetics, development, experience and pathology. Language ability is particularly interesting in this regard, since, at least in monolinguals, it is predominantly left lateralised ( Knecht *et al.*, 1998a). The representation of language in the bilingual brain has been a topic of controversies. While cases of differential recovery patterns for individual languages in stroke patients point towards separate neural representations, neuroimaging of healthy individuals has mostly reported the involvement of overlapping cortical areas for both first and second languages ( Abutalebi *et al.*, 2005; Paradis, 2004).

The neural organisation of language in multilinguals is affected by age of acquisition, proficiency and exposure effects (for neuroimaging and behavioural reviews see Abutalebi *et al.*, 2005; Hull & Vaid, 2007; Perani & Abutalebi, 2005). Typically, differential activation has been reported for late acquisition or low proficiency groups, though the impact of these individual differences seems to be task dependent ( Dehaene & Cohen, 1997; Kim *et al.*, 1997; Klein *et al.*, 1995; Klein, 2003; Wartenburger *et al.*, 2003).

A range of methods has been used to assess anatomical and functional differences between cerebral hemispheres, and depending on experimental aims as well as environmental and task constraints, some methods may be more favourable than others. Here, our focus was on comparing cerebral lateralisation for first and second languages using functional transcranial Doppler ultrasonography (FTCD). This method uses ultrasound to measure cerebral blood flow velocity (CBFV) in the left and right hemispheres. The change in CBFV reflects the task dependent contribution of each hemisphere due to neurometabolic coupling i.e. brain areas showing task-dependent neuronal firing need to replenish metabolic resources, requiring increased blood flow ( Aaslid *et al.*, 1982; Deppe *et al.*, 2004). In order to assess language lateralisation, the middle cerebral artery (MCA), which supplies extensive regions of the cortex, including frontal, temporal and parietal areas, is insonated ( van der Zwan *et al.*, 1993). These cortical regions in the left hemisphere contain areas that are necessary for language processing and production, including Broca’s and Wernicke’s areas in the inferior frontal and superior temporal lobes, respectively. FTCD is a reliable and valid measure of language lateralisation, giving good correlations with the gold standard intracarotid amobarbital test and functional MRI (fMRI) ( Bishop *et al.*, 2009; Deppe *et al.*, 2004; Groen *et al.*, 2012; Illingworth & Bishop, 2009; Knake *et al.*, 2003; Knecht *et al.*, 1998a; Knecht *et al.*, 1998b; Rihs *et al.*, 1999; Somers *et al.*, 2011; Stroobant *et al.*, 2011).

Compared to other methods used to determine lateralisation, FTCD offers a variety of advantages; it is inexpensive, non-invasive, comfortable, easily applicable, mobile and child-friendly ( Bishop *et al.*, 2010; Knecht *et al.*, 1998b). This makes it useful for repeated assessment of language lateralisation. While FTCD has been used to study cerebral lateralisation in monolinguals, it has not, to our knowledge, been used to compare lateralisation of two languages in bilingual participants, i.e. people who use more than one language on a regular basis ( Grosjean, 1989).

In the current experiment, we used the cued word generation task, which is a well validated and commonly used productive language task ( Knecht *et al.*, 1998a; Knecht *et al.*, 1998b), to test whether there are differences in language lateralisation between first (L1) and second (L2) languages in highly proficient bilinguals. A secondary aim was to establish whether lateralisation of L2 was influenced by language history variables, such as age of acquisition. We predicted that the extent of left lateralisation of bilingual speakers would relate to their proficiency levels. In addition, our bilingual participants were native speakers of either French or German, making it possible to see whether there was any indication of language-specific effects, as might be expected if language representation were affected by a similarity between L1 and L2. For instance, on a measure of lexical similarity, English has 60% similarity with German, but only 27% with French ( https://www.ethnologue.com/language/eng).

## Method

### Participants

Participants were recruited through the Oxford University German Society and Oxford University French Society, as well as through posters in the Experimental Psychology building. Participants were aged over 18 years and were either German-English or French-English bilinguals. All had normal or corrected to normal vision. Individuals with a diagnosis of any speech, language or learning impairment, affected by a neurological disorder or taking medication affecting brain function e.g. antidepressants, were not included in the study. A total of 40 individuals were assessed for viability as study participants. In total, 14 participants were excluded for a range of reasons, including no suitable Doppler signal, due to the inability to find a suitable temporal window in the skull, or failure to stabilize the Doppler signal for the required amount of time (11 participants), or low quality data (3 participants). The final sample consisted of 26 individuals (M age = 22.81 years, SD = 3.63; 19 women, 7 men), of whom 11 reported French as their L1 and 15 reported German as their L1.

### Ethics statement

The study was approved by the University of Oxford Central Research Ethics Committee (CUREC), approval number, MS-IDREC-C1-2015-126). All participants provided written informed consent.

### Apparatus

A commercially available transcranial Doppler ultrasonography device (DWL, Multidop T2; manufacturer, DWL Elektronische Systeme, Singen, Germany) was used for continuous measurements of the changes in CBFV through the left and right MCA. The MCA was insonated at ~5 cm (40–60 mm). Activity in frontal and medial cortical areas, supplied by the anterior cerebral artery, and inferior temporal cortex, supplied by the posterior cerebral artery, do not contribute to the measurements made in the MCA. Two 2-MHz transducer probes, which are relatively insensitive to participant motion, were mounted on a screw-top headset and positioned bilaterally over the temporal skull window ( Deppe *et al.*, 2004).

### Handedness

Handedness was assessed via the Edinburgh Handedness Inventory (EHI; Oldfield, 1971). The inventory consists of 10 items assessing dominance of a person’s right or left hand in everyday activities. Each item is scored on a 5 step scale (“always left”, “usually left”, “both equally”, “usually right”, “always right”). A person can score between -100 and +100 for each item and an overall score is calculated by averaging across all items (“always left” -100; “usually left” -50; “both equally” 0).

### Language history questionnaire

The Language Experience and Proficiency Questionnaire (LEAP-Q; Marian *et al.*, 2007) was used to assess language history for all participants. The LEAP-Q is a self-assessment questionnaire consisting of nine general questions and seven additional questions per language that explore acquisition history, context of acquisition, present language use, and language preference and proficiency ratings across language domains (speaking, understanding and reading) as well as accent ratings. Rating scales either involve preference ordering (e.g. please list all the languages you know in order of dominance), stating age or specifying time in years (e.g. age when you began acquiring a language; please list the number of years and months spent in each language environment), or ratings on a scale of 0 to 10. Numeric ratings were used to assess proficiency (0 “no proficiency” to 10 “perfect proficiency”), learning influences (0 “not a contributor” to 10 “most important contributor”), exposure (0 “never” to 10 “always”) and accent (0 “none” to 10 “pervasive”).

The complex range of responses to the LEAP-Q was reduced to six core measures. A general proficiency measure was obtained by averaging the scores for understanding, speaking and reading for L2. Age of acquisition for L2 was assessed by the parameter “age started learning language”. A general measure of accent was calculated averaging across “accent perceived by self” (0 “none” to 10 “pervasive”) and “accent identified by others” (0 “none” to 10 “pervasive”) for L2 (M = 3.08; SD = 2.66). The time spent in an L2 country was used as a measure of immersion for L2. For both learning of and exposure to L2, participants were asked to rate how much factors contributed to learning or are part of their current language exposure (0 “no relevance” to 10 “extreme relevance”); we used the report based on “friends”, since participants reported friends as playing an important role in L2 acquisition and exposure.

### Word generation task

Tasks were programmed using Presentation ^®^ software (version 17.2; www.neurobs.com). All instructions were presented centrally in white Arial font on a black background. Each participant was tested in English (L2) and their native language (L1; French or German) in a single session using two tasks, each consisting of 23 trials.

The order of the two language tasks was counterbalanced across participants and the entire testing session lasted between 75 and 90 minutes. The experimenter spoke English at all times. So that they were focussed on their native language, participants were asked to describe the Cookie Theft picture of the Boston Diagnostic Aphasia Examination in their native language prior to being tested in that language ( Goodglass & Kaplan, 1983).

The cued word generation paradigms were based on Knecht and colleagues’ 1998 paradigm ( Knecht *et al.*, 1998b). For each trial, the participant is shown a letter and is asked to silently generate words starting with that letter. Each task comprised 23 trials and lasted for around 20 minutes. We excluded the three letters with the lowest first letter word frequency: Q, X and Y in English; Q, X and Z in German; and W, X and Y in French. Task instructions for the German and French word generation tasks were translated into German and French by the experimenter.

**Figure 1.**
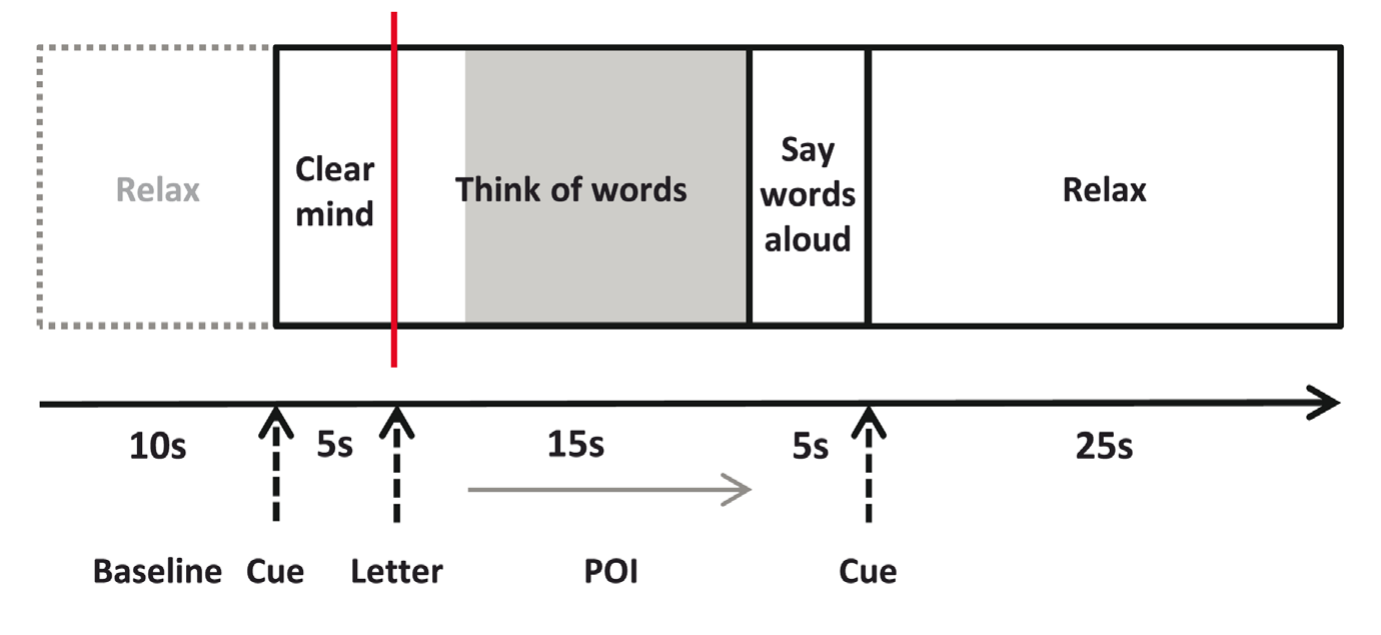
Word generation task. A schematic diagram of the word generation task is depicted. Period of interest is marked in grey from 8 to 20 s, and the event marker is displayed in red. POI, period of interest.

Each trial started with an auditory tone and the written instruction “Clear Mind” (5s), followed by the letter cue to which the participant silently generated words (15s), and then overt word generation (5s) ( Figure 1). To restore baseline activity, participants were instructed to relax (25s) at the end of each trial. Event markers were sent to the Multi-Dop system when the letter cue appeared, denoting trial onset for subsequent analysis of the Doppler signal.

### Data pre-analysis and calculation of asymmetry indices

The blood flow data recorded from the Doppler probes were down-sampled (25 Hz) and analysed using a custom version of the analysis program developed for word generation tasks (DopOSCCI; Badcock *et al.*, 2012a) running in Matlab R2014a ( https://uk.mathworks.com/products/matlab/whatsnew.html; Mathworks, Natick, MA, USA). FTCD data were imported in text format with each column representing one data channel. The data were segmented into epochs, starting at baseline and ending after 30s. Trial baseline was set 10s prior to the cue tone i.e. 15s prior to letter onset ( Figure 1). Following procedures developed by Deppe *et al.* (2004), the data were corrected for insonation angle, physiological changes unrelated to the task, and movement artefacts. The mean of each epoch was used to normalise the data to an arbitrary value of 100 to allow comparison across epochs, and the data were baseline corrected to eliminate slow spontaneous changes in CBFV ( Deppe *et al.*, 2004). The analysis program further included a function for heart cycle integration ( Deppe *et al.*, 2004; Badcock *et al.*, 2012b), artefact rejection and an extra correction to interpolate the signal during very brief periods of dropout or spiking; this could be used once per trial.

Epochs were rejected when the normalised Doppler velocity was extremely high (>140cm/s) or extremely low (< 60cm/s), as these were considered to be measurement artefacts. Epochs were also rejected when values were more than three standard deviations below or four standard deviations above channel mean. Additionally, epochs were manually excluded when issues during the testing session had been recorded (e.g. participant speaking during rest period). The period of interest (POI) was defined between 8 and 20s after cue tone i.e. 3–15s after letter appeared ( Figure 1). For each language, normalised left minus right difference values were determined. This is usually referred to as a ‘laterality index’, but here we use the term ‘asymmetry index’ to avoid confusion between the abbreviations LI (for laterality index) and L1 (for first language). Asymmetry indices were calculated as the mean left-right difference across a 2-s window centred on the maximum peak difference within the POI. Positive values indicated left lateralisation, while negative values indicated right lateralisation.

### Statistical analysis

All statistical data analyses were performed using SPSS version 22.0 (IBM Corp., Armonk, NY).

## Results

Parametric inferential statistics were used for the statistical analyses of the asymmetry indices, since they were distributed normally. Individual differences in data were analysed using non-parametric correlations. Overall, the statistical analyses consisted of analysis of variance, paired samples and independent samples t-tests, and parametric as well as non-parametric correlational analyses.

### Asymmetry indices

**Figure 2.**
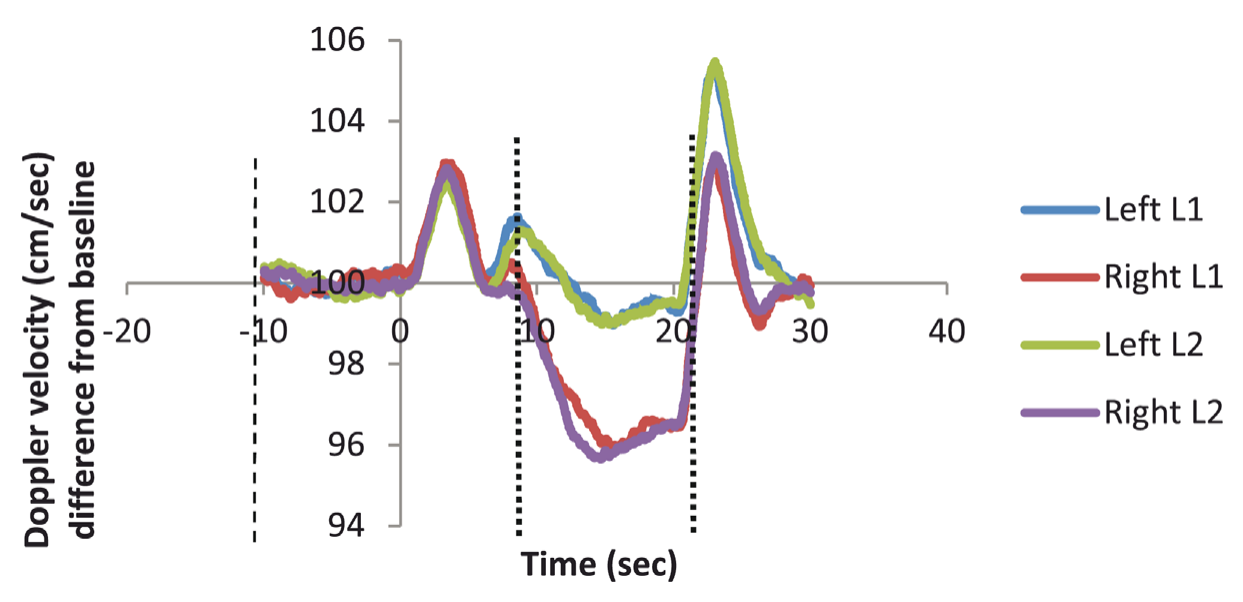
Left and right velocity L1 and L2. Left and right hemisphere activation is displayed at a function of epoch time in seconds for word generation task L1 and L2. Dotted lines indicate the start of the baseline period (left) depicted from -10 to 0 seconds and period of interest from 8 (middle line) to 20 seconds after cue onset (right line). L1, first language; L2, second language.

**Figure 3.**
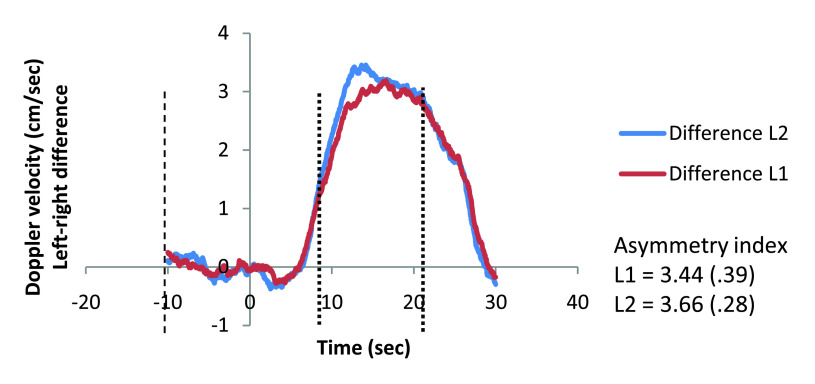
Left-right difference for L1 and L2. Left-right difference in activation is displayed at a function of epochs in seconds for word generation task L1 and L2. Mean asymmetry indices are displayed with standard error in brackets. Baseline period depicted from -10 to 0 seconds and period of interest depicted from 8 to 20 seconds after cue onset. L1, first language; L2, second language.

Normalized Doppler velocities for the left and right hemisphere activation, as well as left minus right differences, are presented for each task in Figure 2 and Figure 3. Descriptive statistics for the general Doppler data are presented in Table 1.

**Table 1.** Number of included trials and asymmetry indices for L1 and L2. Descriptive statistics for the general Doppler data are presented: the number of included trials (out of 23) and asymmetry indices for first and second language. L1, first language; L2, second language; N Trials, number of included trials; Min, minimum; Max, maximum; SEM, standard error of the mean.

Measure		L1	L2
**N Trials**	Mean Min Max	22.42 19 23	22.08 20 23
**Asymmetry index**	Mean SEM	3.44 .39	3.66 .28

**Figure 4.**
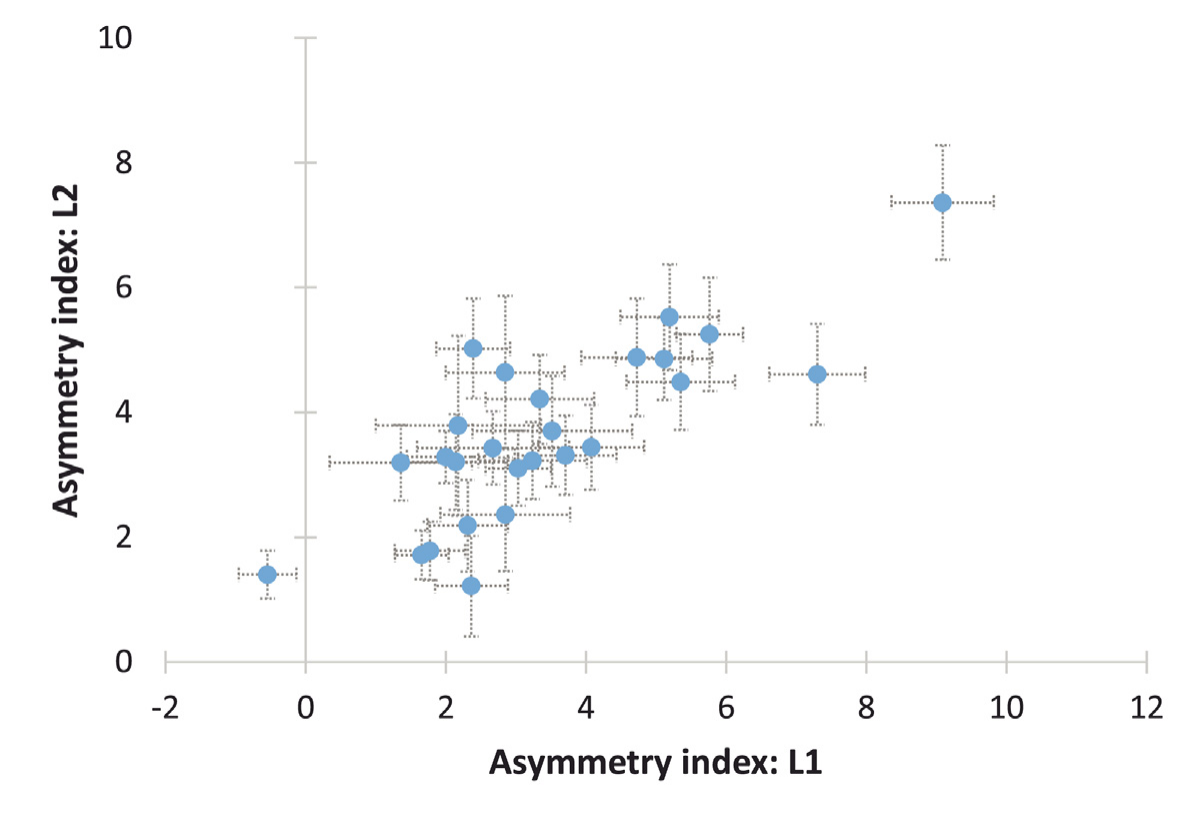
Scatterplot asymmetry indices L1 and L2. Scatterplot displaying the relationship between asymmetry indices for L1 and L2 word generation tasks. Error bars indicate 95% confidence intervals. L1, first language; L2, second language.

At the group level, both language versions of the word generation task produced significantly left-lateralised blood flow. Participants were categorised as left-lateralised if the mean asymmetry index was positive and right-lateralised if the mean asymmetry index was negative or the 95% confidence interval did not span zero. If the 95% confidence intervals crossed zero, language was considered to be bilaterally represented. There were 22 participants categorised as left-lateralised for both L1 and L2, three participants categorised as bilateral for L1 and left-lateralised for L2, and one participant categorised as left-lateralised for L1 and bilateral for L2 ( Figure 4).

Asymmetry indices were analysed using a 2×2 mixed ANOVA with language (L1 vs. L2) as within-subjects factor and first language (German vs. French) as between-subjects factor. The means and standard errors for all dependent measures are shown in Table 2. The main effects of language and first language were not significant; for L1/L2: F (1, 24) = 1.12; p = 0.352; French/German: F (1, 24) = 1.80; p = 0.193. The interaction between these factors was also nonsignificant: F (1,24) = 0.9; p = 0.352. Thus, there was no difference in the asymmetry indices between L1 and L2 for either French or German speakers.

**Table 2.** Asymmetry indices (mean and SEM) for L1 and L2 word generation task separated by first language. Descriptive statistics of asymmetry indices for first and second language word generation tasks separated by first language are presented. Asymmetry indices were analysed using a 2×2 mixed ANOVA with language (L1 vs. L2) as within-subjects factor and first language (German vs. French) as between-subjects factor. Main effects and interaction effects were not significant. L1, first language; L2, second language; N, number of participants; M, mean, SEM, standard error of the mean.

First language	*N*	L1		L2	
		*M*	*SEM*	*M*	*SEM*
**German**	15	3.90	.51	3.93	.37
**French**	11	2.82	.59	3.30	.43

Asymmetry indices for L1 and L2 were significantly positively correlated (Pearson’s: *r* = 0.81; *p* < 0.001; *n* = 26; Figure 4).

To check the reliability of the asymmetry index, Pearson’s product moment correlations between odd and even trials for L1 and L2 were calculated. Odd and even trials for L1 were significantly positively correlated ( *r* = 0.74; *p* < 0.001; *n* = 26). However, odd and even trials for L2 were not significantly correlated ( *r* = 0.28; *p* = 0.162; *n* = 26).

Independent sample t-tests were run to assess the role of testing order and compare asymmetry indices between L1 tested first and L2 tested first for L1 and L2; in neither case was the difference significant ( Table 3).

**Table 3.** Asymmetry indices (mean and SEM) for L1 and L2 word generation task, separated by order of testing. The role of testing order was assessed with two independent sample t-tests, comparing asymmetry indices between L1 tested first and L2 tested first, for L1 (German or French) and L2 (English); in neither case was the difference significant. L1, first language (German or French); L2, second language (English); N, number of participants; SEM, standard error of the mean.

Testing Order	*N*	L1		L2	
		*M*	*SEM*	*M*	*SEM*
**L2 tested first**	12	2.94	.49	3.52	.38
**L1 tested first**	14	3.87	.59	3.78	.41

### Handedness

The handedness measures from the EHI ranged from -22.73 to 100 (M = 73.43; SE = 5.05). Only one participant was left-handed for writing. The handedness score was not correlated with the asymmetry index for either language, nor for the difference in asymmetry indices between languages (absolute values for Spearman correlations were all <0.15).

### Language history

Self-reported measures of language history, as assessed by the LEAP-Q, are displayed in Table 4.

**Table 4.** Means, SEM and ranges for language history measures from LEAP-Q. Descriptive statistics of the six core measures of the LEAP-Q we selected and computed are presented. High mean score for “general proficiency” and early mean age for “age started learning second language” are evident. SEM, standard error of the mean; L2, second language; yr; years; LEAP-Q, Language Experience and Proficiency Questionnaire.

	Mean	SEM	Range
General proficiency a	9.09	0.19	6.3 to 10
Age started learning L2 (yr)	7.62	0.83	0 to 15
Accent b	3.08	0.52	0 to 8
Time spent in L2 country (yr)	4.93	1.04	0.3 to 21
Learning (friends) c	8.04	0.57	1 to 10
Exposure (friends) c	8.00	0.44	0 to 10

a Range: 0 (none) to 10 (perfect)

b Range: 0 (none) to 10 (pervasive)

c Range 0 (no relevance) to 10 (extreme relevance)

The data were tested for normality using the Shapiro-Wilk test. Non-normality was significant for a variety of questionnaire measures, but was not significant for either asymmetry index.

Table 5 shows Spearman’s correlations to explore the relationship between individual differences and asymmetry indices.

**Table 5.** Spearman’s correlations between asymmetry and language history measures (N = 26). Spearman’s correlations to explore the relationship between individual differences and asymmetry indices were carried out. None of the correlations approached significance at Bonferroni-corrected significance level of 0.05/18= 0.002. L1, first language; L2, second language.

	Asymmetry: L1	Asymmetry: L2	Asymmetry difference between L1 and L2
**L2**			
Proficiency (general)	.00	-.01	.10
Age (started learning)	-.08	-.02	.09
Accent (general)	-.02	-.01	-.11
Immersion (country)	.07	.14	-.05
Learning (friends)	-.24	.00	.34
Exposure (friends)	-.35	-.13	.35

None of the correlations between asymmetry indices and individual differences approached significance at a Bonferroni-corrected significance level of 0.05/18 = 0.002.

## Discussion

We had anticipated that language lateralisation might be stronger for a bilingual person’s first language than for the second language, especially for those who were less proficient or who learned the second language later, but this was not confirmed. Nearly all participants showed significant left lateralised blood-flow for both L1 and L2 during the word generation task; only four participants were classified as bilateral for one language, and for three of these it was L1 that was bilateral.

Furthermore, asymmetry indices for L1 and L2 were highly related and similar in magnitude, suggesting good reliability of the measure. When the two languages were considered separately, the correlation between odd and even trials was substantial for L1, but less for L2 asymmetry indices. At first glance this suggests that lateralisation may be more labile in a second language, but we suspect that a more prosaic explanation is likely, i.e. that this result reflects poor quality of the signal in a subset of participants/trials. The odd/even statistic is derived from around 10 trials per condition, and is vulnerable to the effects of outliers. If L2 lateralisation genuinely was more labile, we would not have expected to see the good agreement between the L1 and L2 asymmetry indices when all 23 trials were considered.

We also failed to find any support for the hypothesis that language proficiency affected either absolute levels of language lateralisation or the difference in lateralisation between the two languages. However, this could be due to the level of proficiency in our participants, which was generally very high. Additionally, handedness and asymmetry indices were unrelated. Although in large populations, these variables are related, the association is not strong and it is not uncommon to fail to observe it in small samples ( Bishop *et al.*, 2009).

### Limitations

***1. Sample population and task type.*** Studies using behavioural measures of lateralisation have found that age of acquisition and proficiency are influential factors; early bilinguals tend to show bilateral involvement for both languages, while late bilinguals are left lateralised ( Hull & Vaid, 2007). Our study appears to contradict this finding, but it should be noted that our sample only displays a limited range of age of acquisition and proficiency, raising the possibility that we may have replicated associations between asymmetry and language proficiency or age of acquisition with a more heterogeneous sample.

In addition, we used only one language task, the word generation task. This has been regarded as the gold standard task for FTCD studies, but it may not be optimal for uncovering inconsistent lateralisation between languages. Previous studies have found that the differential impact of age of acquisition, proficiency and exposure effects is task dependent ( Dehaene & Cohen, 1997; Kim *et al.*, 1997; Klein *et al.*, 1995; Klein *et al.*, 2003; Wartenburger *et al.*, 2003). The productive word generation task may be regarded as taxing lexical-semantic processing and fMRI studies have shown that during a lexical-semantic sentence judgement task, only low proficiency individuals display greater activation in language associated areas independent of age of acquisition ( Wartenburger *et al.*, 2003). However, the literature is not consistent on this point, and other studies identify contrasting conclusions, revealing exposure dependent differences in activation during fluency tasks in high proficiency early bilinguals ( Abutalebi *et al.*, 2005; Perani *et al.*, 2003).

In future work, laterality and multilingualism need to be examined through different tasks because both are multidimensional constructs ( Bishop *et al.*, 2013). When other tasks assessing different language components have been used with FTCD, variations in strength or direction of lateralisation became apparent in monolinguals ( Badcock *et al.*, 2012b; Bishop *et al.*, 2009; Haag *et al.*, 2010; Stroobant *et al.*, 2009). The multidimensionality of lateralisation, in particular for language, is suggested by the fact that individual tasks have higher split-half reliabilities than inter-correlations between asymmetry indices from different tasks ( Bishop *et al.*, 2009; Bishop, 2013).

We found no difference in laterality patterns between French-English and German-English bilinguals, but it is possible that differences might be more apparent with languages that are more different from one another, in grammatical structure, lexical items and/or phonology. These factors have been shown to influence the ease with which a second language is learned, and might plausibility affect the extent to which language representations might be shared or distinct ( Schepens *et al.*, 2016).

***2. Language assessment.*** We used a self-report questionnaire to describe our sample and assess language history and proficiency, but behavioural measurements of proficiency may have revealed a wider response range for correlational analysis. Although Marian and colleagues established high reliability and validity for the self-report questionnaire used here, and validated it against behavioural measures, their questionnaire was devised to describe a population rather than provide an analysis measure of individual differences ( Marian *et al.*, 2007).

***3. Method.*** While test-retest reliability of FTCD measurements is high and the time-locked correlation analysis of CBFV is robust and non-invasive, the main limitation of the method is that findings can only be interpreted on a hemispheric level, and do not give the level of localisation possible with other imaging methods ( Somers *et al.*, 2011). To clarify whether the same brain regions within a hemisphere are involved for processing first and second languages, we need measures that provide finer-grained information about within-hemisphere localisation, such as fMRI ( Abutalebi & Green, 2007).

### Conclusion and outlook

Participants were significantly left lateralised for both L1 and L2 when laterality was measured using FTCD during modified versions of the well validated word generation task. The high proficiency group of German-English and French-English bilinguals showed no significant difference between asymmetry indices for L1 and L2, and asymmetry indices were unrelated to language history measures and handedness. However, since laterality and language are multidimensional constructs, FTCD should be used to test bilingual laterality with different tasks and a larger, more heterogeneous sample. As an inexpensive, non-invasive, comfortable, easily applicable, mobile and child-friendly method, with a high temporal resolution, FTCD facilitates testing of large samples, tracking changes throughout development and repeated administration with different tasks. The advantages offered by FTCD are particularly interesting for research on bilinguals, which can therefore potentially outweigh its drawbacks, depending on the research question and study constraints. Furthermore, using FTCD for large-scale assessment of bilinguals could be used to interpret relationships between other cognitive functions and language. However, since FTCD cannot be used to localise specific areas related to changes in functional responses, any conclusions related to localisation have to be drawn carefully and need to be interpreted with reference to other methods.

## Data availability

Open Science Framework: Bilingual FTCD, doi: 10.17605/OSF.IO/VD9DT ( Bishop *et al.*, 2016)

Please see the Data Dictionary for a description of the files.
