# Cerebral lateralisation of first and second languages in bilinguals assessed using functional transcranial Doppler ultrasound

**DOI:** 10.12688/wellcomeopenres.9869.2

**Published:** 2021-07-28

**Authors:** Dorothy V. M. Bishop, Clara R. Grabitz, Sophie C. Harte, Kate E. Watkins, Miho Sasaki, Eva Gutierrez-Sigut, Mairéad MacSweeney, Zoe V. J. Woodhead, Heather Payne

**Affiliations:** 1 Department of Experimental Psychology, University of Oxford, Oxford, UK; 2 Deafness, Cognition, Language Research Centre, UCL, London, UK; 3 Faculty of Business and Commerce, Keio University, Tokyo, Japan; 4 Department of Psychology, University of Essex, Colchester, UK; 5 Institute of Cognitive Neuroscience, UCL, London, UK

**Keywords:** Laterality, Bilingualism, FTCD

## Abstract

**Background**: Lateralised language processing is a well-established finding in monolinguals. In bilinguals, studies using fMRI have typically found substantial regional overlap between the two languages, though results may be influenced by factors such as proficiency, age of acquisition and exposure to the second language. Few studies have focused specifically on individual differences in brain lateralisation, and those that have suggested reduced lateralisation may characterise representation of the second language (L2) in some bilingual individuals.

**Methods**: In Study 1, we used functional transcranial Doppler sonography (FTCD) to measure cerebral lateralisation in both languages in high proficiency bilinguals who varied in age of acquisition (AoA) of L2. They had German (N = 14) or French (N = 10) as their first language (L1) and English as their second language. FTCD was used to measure task-dependent blood flow velocity changes in the left and right middle cerebral arteries during phonological word generation cued by single letters. Language history measures and handedness were assessed through self-report. Study 2 followed a similar format with 25 Japanese (L1) /English (L2) bilinguals, with proficiency in their second language ranging from basic to advanced, using phonological and semantic word generation tasks with overt speech production.

**Results**: In Study 1, participants were significantly left lateralised for both L1 and L2, with a high correlation (r = .70) in the size of laterality indices for L1 and L2. In Study 2, again there was good agreement between LIs for the two languages (r = .77 for both word generation tasks). There was no evidence in either study of an effect of age of acquisition, though the sample sizes were too small to detect any but large effects.

**Conclusion**: In proficient bilinguals, there is strong concordance for cerebral lateralisation of first and second language as assessed by a verbal fluency task.

## Introduction

The two cerebral hemispheres of the brain are neither structurally nor functionally identical. Hemispheric specialisation reflects a variety of factors influencing the brain, including genetics, development, experience and pathology. Language ability is particularly striking in this regard, since, at least in monolinguals, it is predominantly left lateralised in most people ( Knecht *et al.*, 1998a). The representation of language in the bilingual brain has been a topic of controversy. On the one hand, differential recovery patterns for individual languages in stroke patients point towards separate neural representations ( Paradis, 2004), yet on the other hand, neuroimaging of healthy individuals has mostly reported the involvement of overlapping cortical areas in the left hemisphere for first (L1) and second (L2) languages ( Abutalebi *et al.*, 2005; Perani & Abutalebi, 2005; Sulpizio *et al.*, 2020).

The picture is complicated by the complex nature of bilingualism, with individuals varying in age of acquisition (AoA), proficiency, exposure to the different languages, and number of languages spoken. A recent review of brain structure and connectivity concluded that brain organisation was influenced by duration and extent of language use, and their combined effects ( DeLuca *et al.*, 2019). In functional imaging, differential activation for L2 vs L1 has been reported for late acquisition or low proficiency groups, though results have not always been consistent across studies, and the impact of these individual differences appears to be task dependent ( Kim *et al.*, 1997; Klein, 2003; Klein *et al.*, 1995; Wartenburger *et al.*, 2003). More generally, studies on this topic tend to have relatively small sample sizes and hence low power to detect any but large effects.

A range of methods has been used to assess anatomical and functional differences between cerebral hemispheres, depending on experimental aims as well as task constraints. Here our focus is on functional lateralisation, and the possibility that in bilingualism there may be a differential contribution from the right hemisphere for the two languages. This was suggested by a meta-analysis of behavioural studies by Hull & Vaid (2007), incorporating studies using dichotic listening, visual preference, and dual task methods; surprisingly, they found that proficient bilinguals who learned L2 in infancy had more bilateral language representation of L2 than those who acquired L2 after 5 years of age. Few fMRI studies have focussed on language lateralisation in bilinguals. An fMRI study of 16 bilingual people with epilepsy found excellent agreement between laterality indices for L1 and L2 on verb production tasks ( Centeno *et al.*, 2014). In contrast, Dehaene *et al.*, 1997, found, consistent with other studies, that when listening to L1, there was consistency between participants in the locus of activation in the left hemisphere, but when listening to L2, there was substantial variability from person to person, not just within a hemisphere, but also in terms of which hemisphere was most activated. A recent study of basic and advanced L2 learners by Gurunandan *et al.* (2020) reported that, whereas language production tended to be left-lateralised in both languages, in receptive tasks, the two languages tended to lateralise to opposite hemispheres, with this effect increasing with language proficiency. For language production, the size of the laterality index showed only weak agreement between L1 and L2, regardless of proficiency. Taking these findings on language laterality together, we predict that on production tasks, we should find equivalent lateralisation for L1 and L2 in moderate-to-high proficiency bilinguals. Although there is suggestive evidence that laterality indices might show some dissociation between L1 and L2 in bilinguals, this tends to be seen on receptive tasks, and it is hard to know if such dissociations are reliable, as test-retest reliability of the laterality index is usually unknown.

Here we report two studies using functional transcranial Doppler ultrasonography (FTCD) to test the hypothesis that cerebral lateralisation is equivalent for first and second languages in proficient bilinguals. This method uses ultrasound to measure cerebral blood flow velocity (CBFV) in the left and right hemispheres. The change in CBFV reflects the task dependent contribution of each hemisphere due to neurometabolic coupling, i.e. brain areas showing task-dependent neuronal firing need to replenish metabolic resources, requiring increased blood flow ( Aaslid *et al.*, 1982; Deppe *et al.*, 2004). In order to assess language lateralisation, CBFV is measured in the middle cerebral artery (MCA), which supplies extensive regions of the cortex, including frontal, temporal and parietal areas, ( van der Zwan *et al.*, 1993). These cortical regions in the left hemisphere contain areas that are necessary for language processing and production, including classical Broca’s and Wernicke’s areas in the inferior frontal and superior temporal lobes, respectively. FTCD is a reliable and valid measure of language lateralisation, ( Bishop *et al.*, 2009; Groen *et al.*, 2012; Illingworth & Bishop, 2009; Stroobant *et al.*, 2011), giving good correlations with the gold standard intracarotid amobarbital test and functional MRI (fMRI) ( Deppe *et al.*, 2004; Knake *et al.*, 2003; Knecht *et al.*, 1998a; Knecht *et al.*, 1998b; Rihs *et al.*, 1999; Somers *et al.*, 2011). Importantly, FTCD had moderate-to-good within-session (split half) and test-retest reliability ( Woodhead *et al.*, 2020). We can therefore distinguish between true dissociations between LIs on different tasks and lack of agreement attributable to poor reliability of measurement.

FTCD lacks within-hemisphere spatial resolution, so is not suitable for identifying topographic differences in language representation within one hemisphere. However, it provides a measure of changes in blood flow velocity in the middle cerebral artery, which can give a direct index of the relative contribution of the two hemispheres, without any need to specify thresholds or regions of interest. Advantages of FTCD are that it is inexpensive, non-invasive, comfortable, easily applicable, mobile, and child-friendly and it has excellent resolution in the time domain ( Bishop *et al.*, 2010; Knecht *et al.*, 1998b). FTCD has been used to study cerebral lateralisation in monolinguals, but it has not, to our knowledge, been used to compare lateralisation of two languages in bilingual participants, defined here as people who use more than one language on a regular basis ( Grosjean, 1989).

## Study 1: Highly proficient French-English or German-English bilinguals

In Study 1, we used the cued word generation task, which is a well validated and commonly used productive language task ( Knecht *et al.*, 1998a; Knecht *et al.*, 1998b), to test whether language lateralisation is equivalent for first and second languages in bilinguals. A secondary aim was to consider whether there is any impact of AoA. Participants were highly proficient bilinguals, all with English as a second language, who were working or studying in Oxford, UK at an advanced level. We predicted that the extent of left lateralisation of bilingual speakers would relate to their AoA of L2. On the basis of Hull & Vaid's (2007) behavioural meta-analysis we might expect to see weaker lateralisation for L2 in bilinguals with an early AoA. On the other hand, the convergence hypothesis ( Green, 2003) predicts that as proficiency increases, the neural substrate of L1 and L2 become more similar. Green's hypothesis did not focus on lateralisation, but it might nevertheless be taken to suggest the opposite pattern to that predicted by Hull and Vaid, i.e., greater similarity in the neural basis of L1 and L2 in those with the longest experience of L2, i.e. those with early AoA.

### Methods

***Participants*.** Participants were recruited through the Oxford University German Society and Oxford University French Society, as well as through posters in the Experimental Psychology building. Participants were aged over 18 years and were either German-English (N = 14) or French-English (N = 10) bilinguals, with a self-reported high level of proficiency in English. All had normal or corrected to normal vision. Individuals with a diagnosis of any speech, language or learning impairment, affected by a neurological disorder or taking medication affecting brain function e.g. antidepressants, were not included in the study.

A total of 40 individuals were assessed for viability as study participants. In total, 14 participants were excluded for a range of reasons, including no suitable Doppler signal, due to the inability to find a suitable temporal window in the skull, or failure to stabilize the Doppler signal for the required amount of time (11 participants), or low quality data (3 participants). Data was analysed from 26 participants. During the analysis, 2 further participants were dropped because of an insufficient number of useable trials. All further analyses are based on the final sample of 24 participants (18 female; mean age = 23.04 years, sd = 3.64 years).

***Ethics statement*.** The study was approved by the University of Oxford Central Research Ethics Committee (CUREC), approval number, MS-IDREC-C1-2015-126). All participants provided written informed consent.

***Apparatus*.** A commercially available transcranial Doppler ultrasonography device (DWL, Multidop T2; manufacturer, DWL Elektronische Systeme, Singen, Germany) was used for continuous measurements of the changes in cerebral blood flow velocity (CBFV) through the left and right MCA. The MCA was insonated at ~5 cm (40–60 mm). Activity in frontal and medial cortical areas, supplied by the anterior cerebral artery, and inferior temporal cortex, supplied by the posterior cerebral artery, do not contribute to the measurements made in the MCA. Two 2-MHz transducer probes, which are relatively insensitive to participant motion, were mounted on a screw-top headset and positioned bilaterally over the temporal skull window ( Deppe *et al.*, 2004).

***Handedness*.** Handedness was not a selection criterion, and was assessed via the Edinburgh Handedness Inventory (EHI; Oldfield, 1971). The inventory consists of 10 items assessing dominance of a person’s right or left hand in everyday activities. Each item is scored on a 5 step scale (“always left”, “usually left”, “both equally”, “usually right”, “always right”). A person can score between -100 and +100 for each item and an overall score is calculated by averaging across all items (“always left” -100; “usually left” -50; “both equally” 0).

***Language history*.** The Language Experience and Proficiency Questionnaire (LEAP-Q; Marian *et al.*, 2007) was used to assess language history for all participants. The LEAP-Q is a self-assessment questionnaire consisting of nine general questions and seven additional questions per language that explore acquisition history, context of acquisition, current language use, and language preference and proficiency ratings across language domains (speaking, understanding and reading) as well as accent ratings. An overall self-reported proficiency rating was calculated by taking the mean ratings for proficiency in speaking, reading and understanding English.

The main variable of interest from LEAP was age of acquisition of L2 (AoA), i.e. answer to the question ‘age when you began acquiring the language’; we subdivided into early AoA (before 6 years of age) and late AoA subgroups, to test the prediction from Hull & Vaid (2007) that language is more bilaterally represented when L2 is learned in early childhood. To characterise the sample, we also report the numbers of languages spoken; age of achieving fluency in English; self-reported strength of foreign accent when speaking English (on a scale from 0 [none] to 10 [pervasive]); and mean self-reported proficiency in English.

***Word generation task*.** Tasks were programmed using Presentation® software (version 17.2; www.neurobs.com). All instructions were presented centrally in white Arial font on a black background. Each participant was tested in English (L2) and their native language (L1; French or German) in a single session using two tasks, each consisting of 23 trials.

The order of the two languages was counterbalanced across participants and the entire testing session lasted between 75 and 90 minutes. The experimenter spoke English at all times. So that they were focussed on their native language, participants were asked to describe the Cookie Theft picture of the Boston Diagnostic Aphasia Examination in their native language prior to being tested in that language ( Goodglass & Kaplan, 1983).

The cued word generation paradigms were based on Knecht and colleagues’ 1998 paradigm ( Knecht *et al.*, 1998b). For each trial, the participant is shown a letter and is asked to silently generate words starting with that letter. Each task comprised 23 trials and lasted for around 20 minutes. We excluded the three letters with the lowest first letter word frequency: Q, X and Y in English; Q, X and Z in German; and W, X and Y in French. Written task instructions for the German and French word generation tasks were translated into German and French by the experimenter (CG).

**Figure 1.**
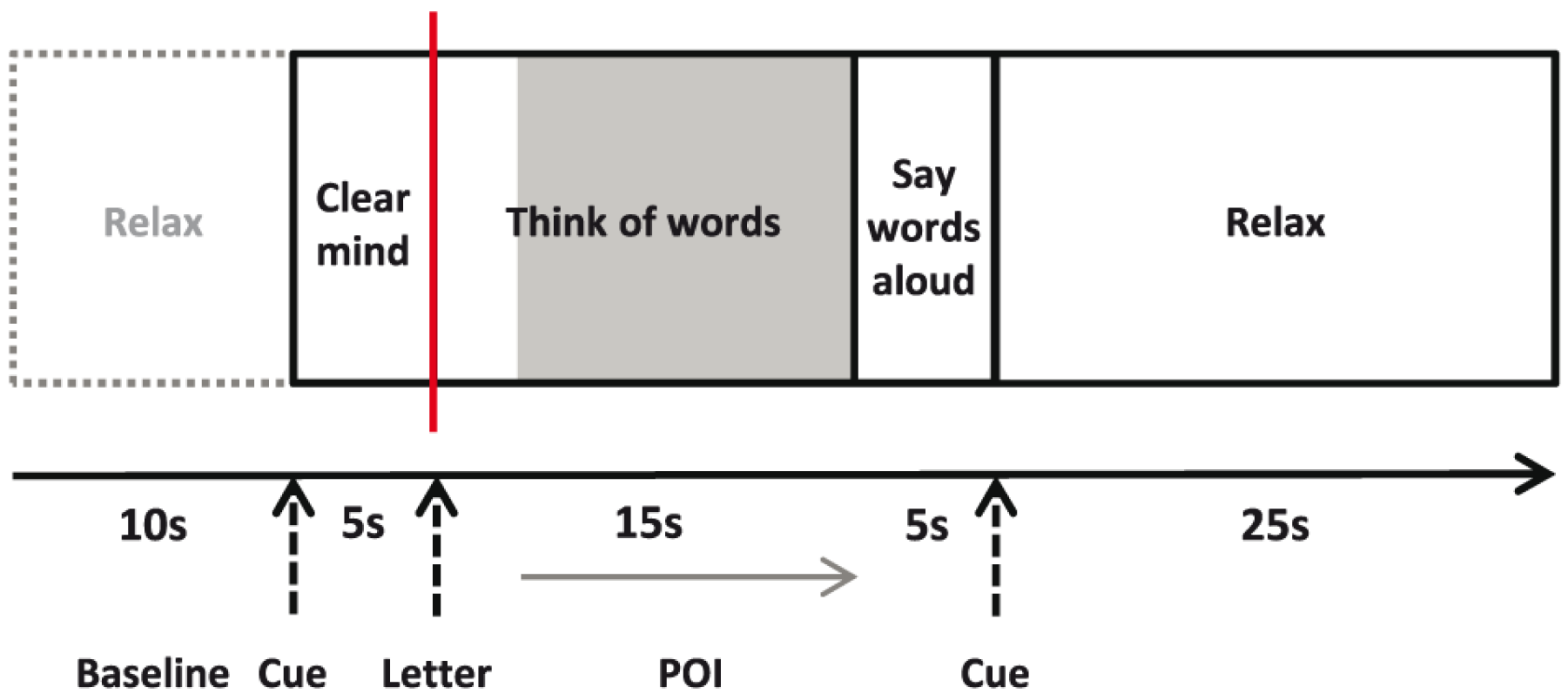
A schematic diagram of the word generation task. Period of interest (POI) is marked in grey from 8 to 20 s, and the event marker is displayed in red.

Each trial started with an auditory tone and the written instruction “Clear Mind” (5 s), followed by the letter cue to which the participant silently generated words (15 s), and then overt word generation (5 s) ( Figure 1). To restore baseline activity, participants were instructed to relax (25 s) at the end of each trial. Event markers were sent to the Multi-Dop system when the letter cue appeared, denoting trial onset for subsequent analysis of the Doppler signal.

***Data pre-analysis and calculation of asymmetry indices*.** The cerebral blood flow velocity data were analysed using custom scripts in R Studio ( R Core Team, 2020), which are available in the *Underlying data* ( Bishop *et al.*, 2021a). The data preprocessing followed conventional methods ( Deppe *et al.*, 2004), and included the following steps:

- Downsampling from 100 Hz to 25 Hz.
- Epoching from -11 s to 30 s relative to the onset of the ‘Clear Mind’ cue.
- Manual exclusion of trials with obvious spiking or dropout artefacts.
- Automated detection of data points with signal intensity beyond 0.0001-0.999 quantiles. If a trial contained one of these extreme data points, it was replaced by the mean for that epoch; if it contained more than one, the trial was excluded from further analysis
- Normalisation of signal intensity by dividing CBFV values by the mean for all included trials and multiplying by 100.
- Heart cycle integration by averaging the signal intensity from peak to peak of the heartbeat.
- Baseline correction by subtracting the mean CBFV across the baseline period (-10s to 0s relative to the ‘Clear Mind’ cue) from all values in the trial.
- Automated detection and rejection of trials containing normalized values below 60 or 140.

Participants with fewer than 15 usable trials for either language were excluded from all further analyses. For each participant that was included in the analysis, a grand mean was calculated over all of their included trials. A laterality index (LI) was calculated by taking the mean of the difference between left and right CBFVs (L-R) within a period of interest (POI) that started 8 s after the ‘Clear Mind’ cue (i.e. 3 s after the word generation task had begun) and ended at 20 s (i.e. when the covert generation task ended). The start time of the POI was chosen to allow time for the blood flow to respond to the task; and the end time was chosen to prevent capturing the response to the overt speech generation phase.

This method of calculating LI using the mean L-R difference across the whole of the POI (the ‘mean’ method) deviates from the conventional method that we had used in the first version of this paper ( https://doi.org/10.12688/wellcomeopenres.9869.1). The original ‘peak’ method, popularised by Deppe *et al*. (1997) takes the mean of a narrow time window around the peak difference within the POI. This method forces the LI to be either left or right - even if the waveform is close to zero with no clear lateralised peak, the highest absolute value in the POI will be treated as a peak. This creates a bimodal distribution of LIs. We have compared the ‘peak’ method with our ‘mean’ method, and shown that, while they give high agreement, the mean method is at least as reliable and gives normally distributed LI values, albeit with lower values, due to averaging over the whole POI ( Woodhead *et al.*, 2020). We have therefore moved to using the mean method in our current research. Nonetheless, peak LI values were computed in case they are required for comparison with other studies, and are available on the online data repository: https://osf.io/4pm76/.

In a final step, to bring our methods in alignment with Woodhead *et al.* (2019), we identified and excluded datasets with unusually high trial-by-trial variability using the Hoaglin & Iglewicz (1987) outlier detection method. For this analysis, LI was calculated for each trial, rather than just for the grand average. The standard error of these single-trial LI values was then calculated. Outliers were defined as datasets where the standard error was above an upper threshold, calculated as:

Upper threshold = Q3 + 2.2 * (Q3 – Q1)

where Q1 is the first quantile of the standard errors among all participants, and Q3 is the third quartile. Participants who had standard error above the upper threshold for either L1 or L2 were excluded from all further analyses.

***Statistical analysis*.** All analyses were conducted using the R Programming Language ( R Core Team, 2020). We first checked for a leftward bias in the overall laterality index, using a one-group t-test, and also categorised each participant as left-biased, right-biased or bilateral. The bilateral group were those whose confidence interval around the LI included zero. Split half reliability of the LI was estimated using LIs computed from odd or even trials only. Spearman correlations were computed between LIs for L1 and L2.

To test our main hypothesis, the association between strength of lateralization (LI values) for L1 and L2 was first visualized using a scatterplot, with the strength of association computed as Spearman’s correlation coefficient. Following Woodhead *et al.* (2020), we adopted an approach based on Bland & Altman (1986) to determine whether the individual LIs for L1 and L2 were equivalent. This involves specifying boundaries for the expected distribution of difference scores, which should contain 95% of bivariate points, if the two values are equivalent. The expected range can be computed from knowledge of the task reliability. We adopted the range specified by Woodhead *et al.* (2020); they computed difference scores by LIs for odd vs even trials, and set boundaries corresponding to expected mean of zero +/-1.96 standard deviations. If the two measures are equivalent, 95% of difference scores, the repeatability coefficient, between LIs for L1 and L2 should fall in this range (from -2.5 to 2.5).

For our second hypothesis, that laterality for L2 would be associated with AoA, we used a t-test to compare laterality for L2 between those with early vs late AoA. A two-tailed test was used because the literature does not give clear predictions about direction of effect.

In addition, we report the correlation between LI values and strength of handedness (EHI quotient), and the impact of testing order (L1 then L2, or L2 then L1).

### Results

***Handedness*.** Summary statistics for the EHI handedness measure can be seen in Table 1. Of 24 participants included in the data analysis, 23 had EHI values above 0, indicating right handedness. The remaining participant had an EHI of -20, indicating weak left handedness. Correlations between LI from FTCD and handedness scores on the EHI, were not statistically distinguishable from zero for either L1 (r = -0.145) or L2 (r = 0.137).

**Table 1.** Demographics for the Study 1 participants, N=24 (18 female).

Characteristic	Mean (sd)
Age, years	23.04 (3.64)
EHI/100	73.67 (26.74)
Languages spoken	3.71 (0.95)
Age of English acquisition, years	7.54 (4.41)
Age of English fluency, years	12 (6.83)
English accent/10	2.58 (2.41)
English overall rating/10	9.1 (1)
English speaking rating/10	8.92 (1.1)
English listening rating/10	9.12 (1.08)
English reading rating/10	9.25 (0.94)

***Language history*.** Summary statistics for the language history questionnaire can be seen in Table 1. Self-reported proficiency in speaking, reading and understanding English were all generally high (all around 9/10), with a minimum for any individual rating of 6/10. Age of acquisition, defined as age when first started acquiring the language, was more variable, ranging from 0 to 15 years. Binary categorisation of AoA, using Hull & Vaid’s (2006) criteria gave 7 cases of early AoA (below 6 years of age), and 17 cases of late AoA.

***FTCD data quality and reliability*.** As mentioned in the Methods, two participants were excluded from the analysis because of insufficient number of usable trials. For the remaining 24 participants, 5.98% of trials were excluded for L1, and 6.34% for L2.

Normality of the LI values was assessed using Shapiro-Wilk tests. Distributions of LIs were unimodal for both L1 and L2. Data for L1 did not significantly deviate from normality (W = 0.88, p = 0.009), whereas data for L2 were significantly non-normal (W = 0.96, p = 0.514), showing a rightward skew.

Split-half reliability was assessed by correlating the LI values from odd and even trials. The Spearman’s correlation for the L1 data was 0.58, and for the L2 data it was 0.7, indicating medium to good within-session reliability.

**Figure 2.**
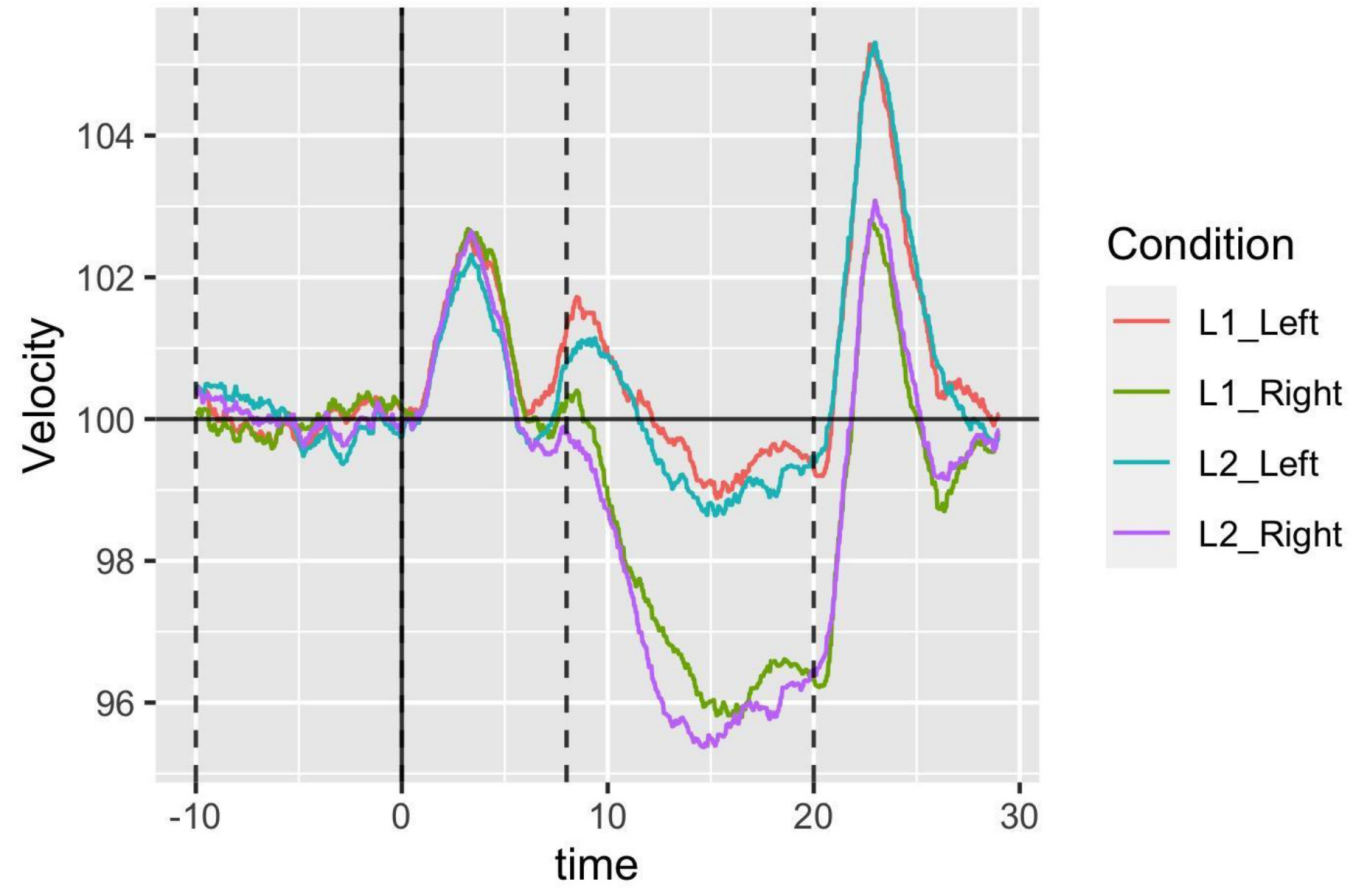
Left and right hemisphere activation is displayed as a function of epoch time in seconds for the word generation task for L1 (French or German) and L2 (English) in Study 1. Dotted lines indicate the start and end of the baseline period (from -10 to 0 seconds) and the period of interest (from 8 to 20 seconds). L1, first language; L2, second language.

Normalized blood flow velocities for the left and right middle cerebral arteries are presented for each task in Figure 2.

Table 2 shows summary statistics for the LI values for L1 and L2. The Bayes factor was computed to check the equivalence of the mean LI for the two languages using the R package ‘BayesFactor’ with default settings ( Morey & Rouder, 2018), and gave a value of 0.234, which may be interpreted as moderate evidence for the null hypothesis ( Lee & Wagenmakers, 2014). The percentage of participants in each group categorised as left lateralised, bilateral or right lateralised is also shown. The majority of participants were left lateralised, with only around 10% showing bilateral activation. No participants showed right lateralisation for either L1 or L2. T-tests showed that there were no significant effects of testing order on LI values, either for L1 (p = 0.113) or L2 (p = 0.712).

**Table 2.** Summary statistics for Study 1 laterality indices (N = 24).

Language	Mean trials	mean LI	se LI	% left	% bilateral	% right
L1	21.62	2.72	0.36	92	8	0
L2	21.54	2.82	0.28	88	12	0

**Figure 3.**
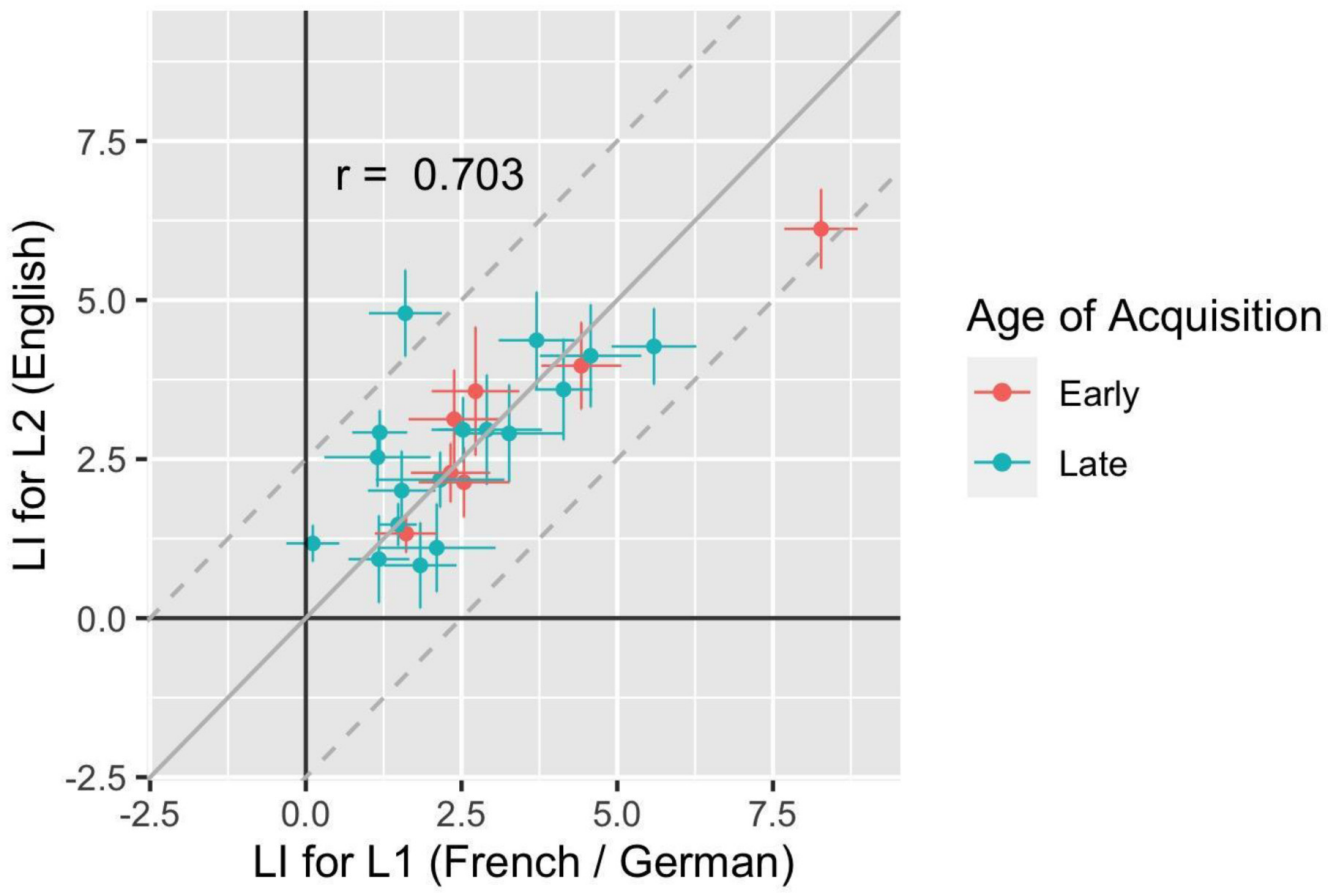
Scatterplot showing individual mean LIs in L1 and L2, with horizontal and vertical error bars denoting standard errors. The continuous grey line corresponds to the point of equality of the two measures, and the dotted lines show the limits where difference between LIs is +/- 2.5.

As can be seen in the scatterplot in Figure 3, laterality indices for L1 and L2 were similar, with Spearman’s R = 0.703. Furthermore, the points cluster around the continuous grey line, which shows the point of equivalence between L1 and L2, and all but one point falls within the Bland-Altman bounds (dotted grey lines), as would be expected if L1 and L2 were equivalent.

***Effect of age of acquisition*.** One can see by inspection of Figure 3 that there is no evidence of a trend for lower LI for L2 in those with early AoA, and a t-test of differences in L2 LI for those with early and late AoA revealed no differences: t = 0.84, p = 0.419. For a more quantitative assessment of association, we computed Spearman’s correlations between the LI values for L2 (English) and the age of acquisition of English. This was not statistically different from zero (r = 0, p = 0.99).

### Discussion

Nearly all participants showed significant left lateralised blood-flow for both L1 and L2 during the word generation task. Only 5 participants were classified as bilateral for one language, and for 3 of these it was L1 that was bilateral. Furthermore, laterality indices for L1 and L2 were highly related and similar in magnitude, indicating good reliability of the measure. Proficiency was generally high in this sample, so it was not possible to assess the impact of variation in proficiency on lateralisation. The sample was small, and so lacking in power to detect small effects, but there was no indication of support for the hypothesis that AoA affected absolute levels of language lateralisation or was related to a difference in lateralisation between the two languages.

## Study 2: Japanese-English bilinguals with moderate-high proficiency

In Study 1 we found no difference in laterality patterns for L1 and L2 between French-English and German-English bilinguals, but it is possible that differences might be more apparent with languages that are more different from one another, in grammatical structure, lexical items and/or phonology. These factors have been shown to influence the ease with which a second language is learned, and might plausibly affect the extent to which language representations are shared or distinct ( Schepens *et al.*, 2016). Study 2 provided the opportunity to assess this idea in a sample of adults whose native language was Japanese, with English as the L2.

Study 2 was run independently of Study 1, at a different institution by different experimenters, to address similar questions to Study 1, but with Japanese-English bilinguals. We report the two studies together here as they make it possible to test generalisability of the Study 1 findings in a different language, and with some methodological modifications. In addition, Study 2 included bilinguals with a wider range of proficiency than Study 1, making it possible to consider the effect of this variable on lateralisation.

An additional aim of Study 2 was to test whether a language that uses both logographic and syllabic orthographic systems would show a more pronounced difference between phonological and semantic processing in the strength of lateralisation (cf, Gutierrez-Sigut *et al.*, 2015). Japanese Kana carry phonological information, but Kanji are more strongly linked to semantic information. We expected that phonological fluency would stimulate typically left-lateralised pre-motor articulatory planning processes more strongly than semantic fluency, and therefore be more strongly left-lateralised.

### Methods

***Participants*.** We recruited participants through the UCL psychology participant pool, research posters around the University, and through email communication to contacts within Japanese communities in London. We initially recruited 32 adult native speakers of Japanese, who reported using English on a daily basis. None of the participants had a history of reading or language difficulties. All had normal or corrected to normal vision.

Seven participants were excluded from the study. This was due to inability to find a suitable temporal window (6 participants), or an insufficient number of usable trials after preprocessing (1 participant). All analyses are based on the final sample of 25 participants] (19 female, mean age = 29.32 years, sd = 6.73 years).

***Ethics statement*.** Ethical approval for the study was granted by the UCL Research Ethics Committee (ID:3612/001). Participants gave written informed consent and were aware they could withdraw at any time.

***Language history and ability*.** Age of acquisition of English and number of years of using English were evaluated via self-report. As with Study 1, a binary age of acquisition (AoA) variable was created by subdividing participants into early (below 6 years) and late (6 years or over) subgroups.

English language ability was measured using the Quick Placement Test ( University of Cambridge Local Examinations Syndicate, 2001), which assesses English reading, vocabulary, and grammar. The test is scored out of 60. Those who scored under 40 were classed as having basic level proficiency (N = 4); between 40 and 48 were classed as having intermediate level proficiency (N = 3); and above 48 were classed as having advanced level proficiency (N = 17). The test data was not available for one participant.

***FTCD apparatus*.** Blood flow velocity through the left and right MCAs was examined using a DopplerBox ultrasonography device and DiaMon headset (manufactured by DWL Elektronische Systeme, Singen, Germany). Two 2-MHz transducer monitoring probes were mounted on the headset and placed at each temporal skull window.

***Word generation tasks*.** Stimuli were presented using Cogent toolbox ( http://www.vislab.ucl.ac.uk/cogent) for MATLAB (Mathworks Inc., Sherborn, MA). Triggers time locked to the onset of the stimulus were sent from the presentation PC to the Doppler Box set-up.

The task was based on Gutierrez-Sigut *et al.* (2015), and involved phonological and semantic word generation tasks in English and Japanese, with order counterbalanced across participants. Task instructions were delivered to correspond to the tested language. Unlike in Study 1, there was no silent interval for covert word generation: participants spoke the words aloud as they thought of them. Gutierrez-Sigut et al. had previously shown that LIs were similar regardless of whether overt or covert responses were given, and they noted a benefit of overt production was that the experimenter could record the participants’ responses as they occurred. For each trial, participants saw “Clear Mind” presented on the screen for 3 seconds. The cue stimulus was then presented, and participants had 17 seconds to overtly generate as many words as possible. Participants were then instructed to relax for 16 seconds to restore baseline activity. Each trial lasted a total of 36 seconds.

***Stimuli***

***Phonological word generation - Japanese and English.*** In Japanese, participants were presented with a cue in Hiragana, one of the Japanese phonological scripts. Following the Japanese mora frequency analysis conducted by Dan *et al.* (2013) based on the familiarity ratings in Amano & Kondo (1999), 10 of the 12 most frequent moras that are positioned at the beginning of words were selected (あ/a/, い/i/, お/o/, か/ka/, き/ki/, こ/ko/, さ/sa/, し/shi/, た/ta/, ふ/hu/). The two moras omitted were は (/ha/) and じ (/ji/). は was omitted because it would be pronounced /wa/ when it was the subject-marker and じ was omitted because it was the voiced sound of し (/shi/) that was included in the stimuli. Participants had to produce as many words as possible that began with the specified Kana. Each Kana was presented twice, and the 20 trials were presented in a pseudo-randomised order to ensure all 10 cues had been presented once before a cue was repeated.

In the English phonological word generation task, participants were presented with 10 alphabetic letters (A, B, C, F, H, M, O, S, T, W) and asked to produce as many words as possible that began with the specified letter. Trials were presented in the same manner as the Japanese task.

***Semantic word generation - Japanese and English.*** Ten Japanese words representing semantic categories were presented in the standard written form, i.e. the mixture of Kanji and Kana: 家畜 farm animals, 動物園の動物 zoo animals, 野菜 vegetables, 果物 fruits, 飲み物 drinks, 色 colours, スポーツ sports, ペット pets, 道具 tools, and 乗り物 transport. The same semantic categories were presented in English. Participants had to report as many words that matched these categories as possible. Each category was repeated twice in the semantic fluency blocks. Categories were presented in a pseudo randomised order.

***FTCD analysis*.** The same FTCD analysis method was used as in Study 1, except that the epoch lengths were changed to match timings for Study 2. The POI started at 6 s after the onset of the ‘Clear Mind’ stimulus (i.e., 3 s after the word generation task had begun) and ended at 20 s (i.e., at the end of the word generation task).

### Results

***Language history and task performance*.** Summary statistics of language history can be seen in Table 3. Age of English acquisition ranged from 0 to 13 years. In contrast to Study 1, where there was little variation in proficiency: Study 2 included 4 cases with basic proficiency, 3 cases with intermediate proficiency, and 17 cases with advanced proficiency, according to the Quick Placement Test. The usage of English was assessed using the question “how much English and Japanese (and other languages if you have) do you use in a typical week?” and the percentages of use of English out of 100% are shown in Table 3. The participants tended to use English more than Japanese.

**Table 3.** Demographics for the Study 2 participants, N=25 (19 female).

Characteristic	Mean (sd)
Age, years	29.32 (6.73)
Age of English acquisition, years	10 (4.21)
Time using English, years	11.08 (6.43)
English overall score/60	47.38 (7.1)
English speaking/100	65.65 (20.96)
English listening/100	69.78 (16.48)
English reading/100	65.43 (16.51)
English writing/100	69.78 (18.8)

The mean number of words produced per trial in the phonological conditions was 5.84 (SD = 1.34) for Japanese and 5.98 (SD = 1.32) for English. The mean number of words produced per trial in the semantic condition was 7.61 (SD = 1.24) for Japanese and 6.95 (SD = 1.29) for English. There was no significant difference between the mean number of words produced per trial for L1 and L2 in the phonological condition (t (48) = -0.36, p = 0.719) or the semantic condition (t (47.9) = 1.84, p = 0.071).

***FTCD data quality and reliability*.** Normality of LI values was assessed using Shapiro-Wilk tests. For the phonological tasks, data was normally distributed for L1 (W = 0.96, p = 0.507) and L2 (W = 0.95, p = 0.278). Data was also normally distributed for the semantic tasks for L1 (W = 0.97, p = 0.63) and L2 (W = 0.98, p = 0.949).

Split-half reliability was assessed by correlating the LI values from odd and even trials, using Spearman’s correlations for consistency with Study 1. For phonological word generation, the split-half correlation was 0.6 for L1 and 0.83 for L2. For semantic word generation, the correlation was 0.61 for L1 and 0.69 for L2. This indicated moderate to good reliability for all tasks.

**Figure 4.**
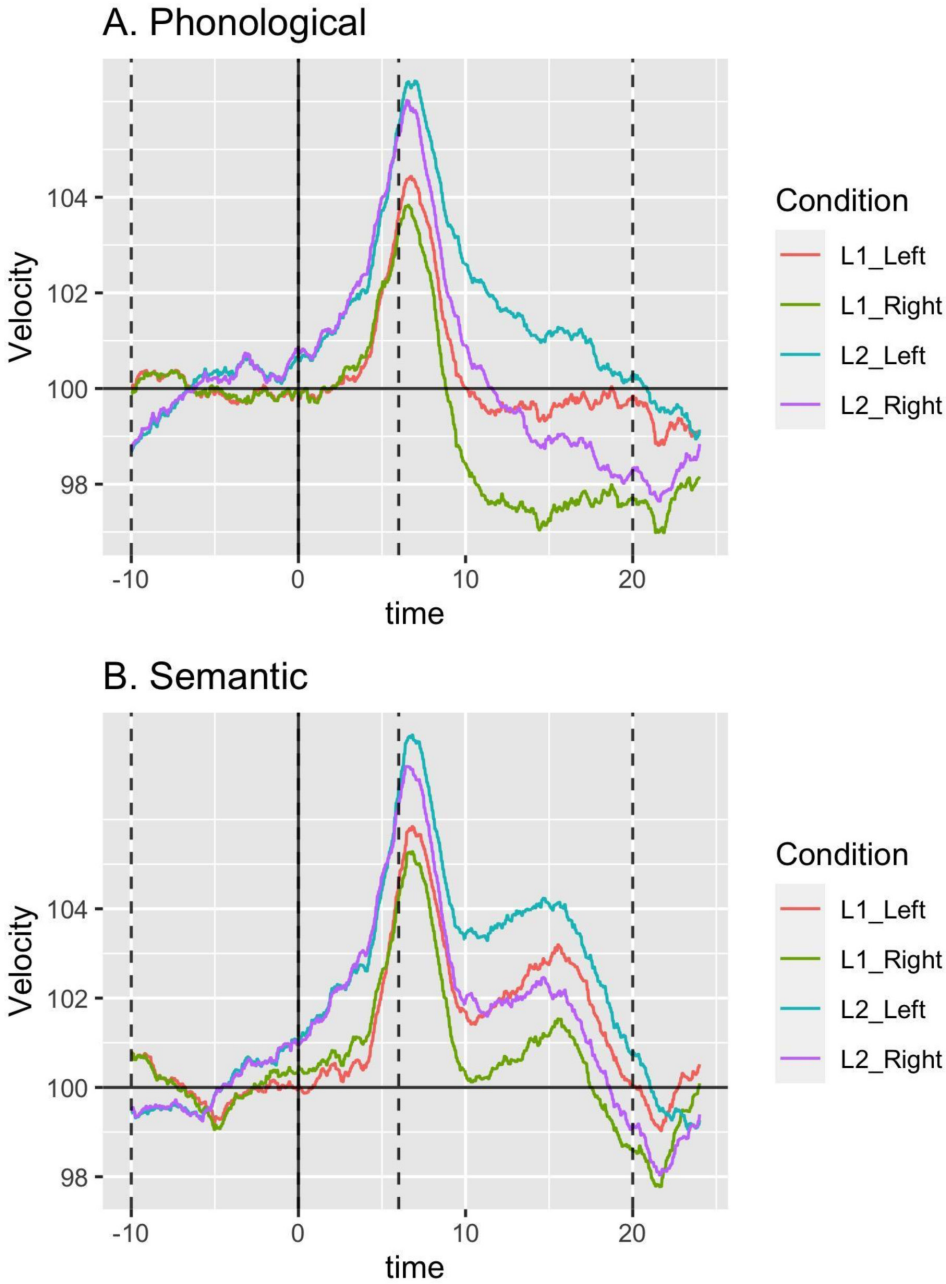
Left and right hemisphere activation is displayed as a function of epoch time in seconds for word generation for L1 (Japanese) and L2 (English) in Study 2. Plot 4a shows the phonological word generation task, and 4b shows the semantic word generation task. Dotted lines indicate the start and end of the baseline period (from -10 to 0 seconds) and the period of interest (from 6 to 20 seconds). L1, first language; L2, second language.

***LI values*.** Normalized blood flow velocities for the left and right middle cerebral arteries are presented for each language and task in Figure 4. Table 4 shows summary statistics for L1 and L2 in both phonological and semantic word generation tasks. Bayes factors were computed to check the equivalence of the mean LI for the two tasks in the two languages using the R package ‘BayesFactor’ with default settings ( Morey & Rouder, 2018). This gave a value of 0.211 for the Phonological task, which may be interpreted as moderate evidence for the null hypothesis, and a value of 0.368 for the Semantic task, which corresponds to anecdoal evidence for the null hypothesis ( Lee & Wagenmakers, 2014).

**Table 4.** Summary statistics for Study 2 laterality indices.

Task	Language	Mean trials	mean LI	se LI	% left	% bilateral	% right
Phonological	L1	19.12	1.96	0.28	72	28	0
Phonological	L2	19.52	2.01	0.38	76	20	4
Semantic	L1	19.00	1.53	0.28	72	28	0
Semantic	L2	19.12	1.65	0.28	64	32	4

**Figure 5.**
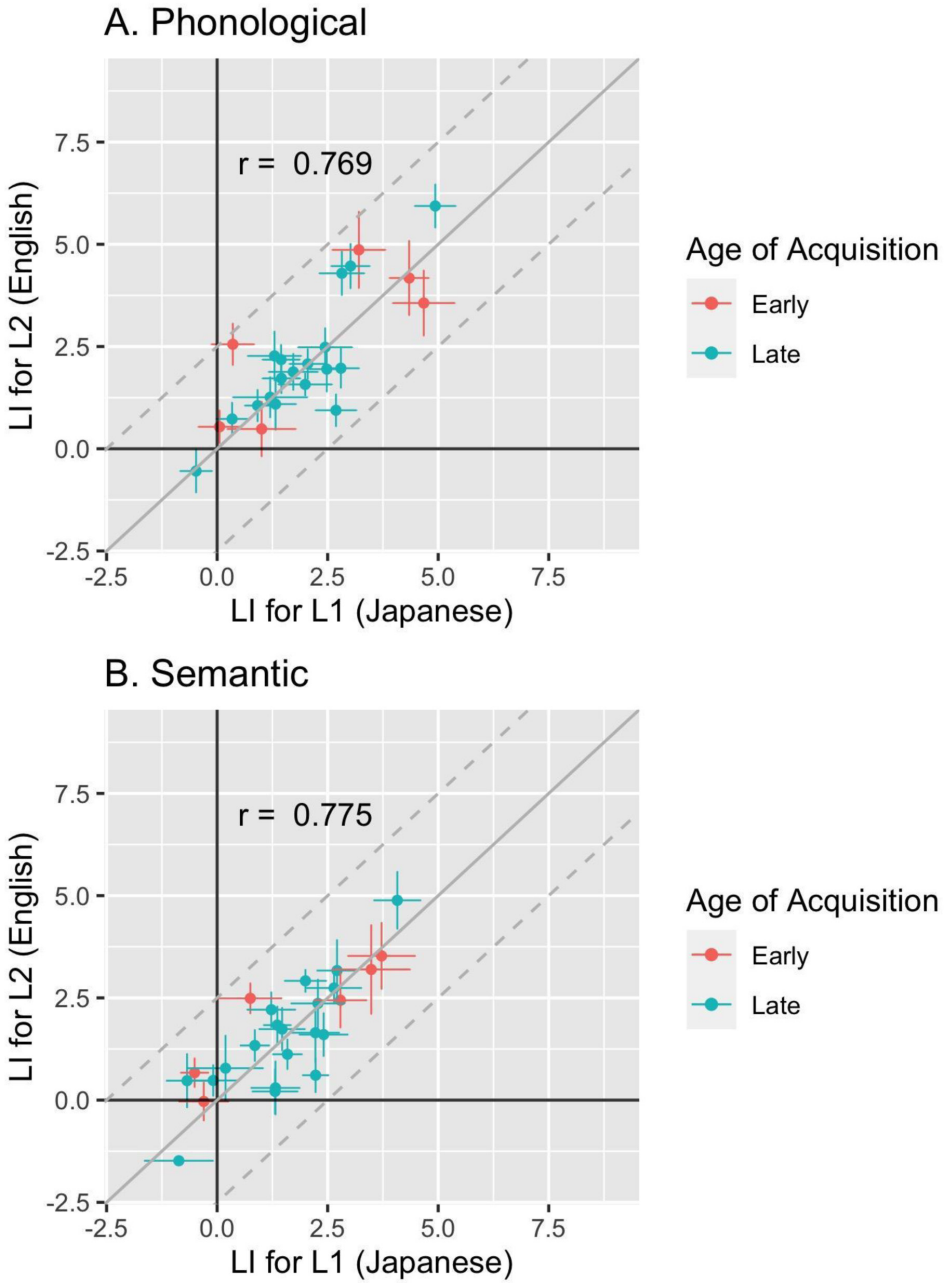
Scatterplot showing individual mean LIs in L1 and L2 for ( **a**) Phonological and ( **b**) Semantic Word Generation, with horizontal and vertical error bars denoting standard errors. The continuous grey line corresponds to the point of equality of the two measures, and the dotted lines show the limits where difference between LIs is +/- 2.5.

Laterality indices for L1 and L2 were strongly correlated in both the phonological task (Spearman’s R = 0.769) and the semantic task (Spearman’s R = 0.775), closely replicating the results of Study 1. This is shown in the scatterplots in Figures 5a and 5b.

***Effects of age of acquisition and proficiency*.** Points in Figure 5 are coded to show age of acquisition. We explored whether age of acquisition for English was related to strength of laterality in L2. There was no significant correlation between AoA and LI for the phonological task (r = -0.12, p = 0.583; Figure 5A) or for the semantic task (r = -0.02, p = 0.907; Figure 5B).

Data on the Quick Placement Test, the measure of proficiency in L2, were available for 24 participants. These were not correlated with the LI for either the phonological task: r = 0.11, p = 0.614, or the semantic task: r = 0.02, p = 0.932.

### Discussion

Study 2 found that most participants were left-lateralised for language on both tasks in both languages and there was close correspondence between the LIs for L1 and L2. Furthermore, the pattern of results was very similar for the phonological and semantic fluency tasks. For this sample we had direct measures of proficiency, but again we found no relationship between lateralisation and either age of acquisition or proficiency.

## General discussion

The results of Studies 1 and 2 show strong similarity despite the differing format of the tasks (covert and overt), native languages (French/ German and Japanese), and English proficiencies (mostly highly proficient but varying between basic and advanced proficiency).

The correlations between the LIs for L1 and L2 were uniformly high (ranging from .70 to .78) with 79% of participants left lateralised for L1 and 76% of participants left lateralised in L2. The data reported here add to a growing pool of results supporting the idea that laterality of expressive language processing is the same for L1 and L2 in proficient bilinguals.

It is worth highlighting that our studies only used expressive language tasks, which typically produce strong lateralisation. Where discrepancies in laterality have previously been reported, this has been for receptive language tasks - both in behavioural contexts (dichotic listening), and in neuroimaging (comprehension or lexical decision tasks) ( Gurunandan *et al.*, 2020; Hull & Vaid, 2007; Wartenburger *et al.*, 2003). It is possible that the processes that drive this effect seen in the literature are not recruited during expressive language production. There would be considerable interest in studying laterality of perception and comprehension of spoken language using FTCD, for which we have developed some paradigms that have good reliability ( Woodhead *et al.*, 2020).

Split-half reliabilities for all tasks were also uniform and high (ranging from .58-.83) for both languages. This suggests that previously reported dissociations between laterality for L1 and L2 could simply reflect low reliability of the chosen measure. We believe our results are not an artefact of bimodality in the distributions; few cases had atypical lateralisation, and we used nonparametric correlations to guard against undue influence on correlations by outliers.

Age of acquisition has been proposed as a key factor in determining divergence of lateralisation patterns. For example Hull & Vaid (2007) found that bilinguals who were exposed to a second language before the age of 5 years had more bilateral representation than those who acquired a second language later. In our studies, age of acquisition (defined as age at first acquiring L2) ranged from 0–15 in Study 1 and 0–13 in Study 2. We found no difference in lateralisation strength for L1 and L2 in those who acquired English early compared to those who acquired English later in either study for both phonological and semantic word generation tasks. This suggests that when a second language is proficiently acquired, lateralisation patterns of expressive language remain stable, regardless of age at which acquisition began.

Our research can also add to the literature regarding lateralisation and proficiency. Study 2 included participants with proficiency levels varying from basic to advanced as measured by the standardised Quick Placement Test. Gurunandan *et al.* (2020) reported that increasing proficiency of L2 accompanied more divergent lateralization patterns between L1 and L2. This result was not replicated in our study, with participants of all proficiencies showing similar LI strengths across all tasks. As we found no indication that degree of language laterality is of functional significance this opens up the possibility that variations in strength of LI, as measured by FTCD, may reflect anatomical differences. Individual variation in anatomy of the cerebral blood vessels has been documented ( Payne, 2017), but has not, to our knowledge, been related to measures of lateralised blood flow.

### Limitations

***Sample population:*** Our samples were relatively small, with relatively few individuals with early age of acquisition or low proficiency. Given the dearth of data on cerebral lateralisation in bilinguals, we feel that nevertheless, the data are worth reporting so they can contribute to future meta-analyses. To that end we have made the data openly available in a repository.

***Language assessment*:** In study 1, we used a self-report questionnaire to describe our sample and assess language history and proficiency, but behavioural measurements of proficiency may have revealed a wider response range for correlational analysis. Although Marian and colleagues established high reliability and validity for the self-report questionnaire used here, and validated it against behavioural measures, their questionnaire was devised to describe a population rather than provide an analysis measure of individual differences ( Marian *et al.*, 2007).

For study 2, we had a direct measure of language proficiency, but we did not find any coherent associations between level of proficiency and lateralisation.

***Method*:** While test-retest reliability of FTCD measurements is high and the time-locked correlation analysis of CBFV is robust and non-invasive, the main limitation of the method is that findings can only be interpreted on a hemispheric level, and do not give information about brain regions within a hemisphere that are involved for processing first and second languages. To uncover the specific networks involved in processing L1 and L2, we would need techniques that provide finer-grained information about within-hemisphere localisation, microcircuitry, and connectivity ( Abutalebi & Green, 2007).

## Conclusions

In two studies, we showed that proficient bilinguals have comparable levels of lateralisation for L1 and L2 when laterality is measured using FTCD during modified versions of the well-validated word generation tasks. Our results indicate that degree of language laterality is reasonably stable in individuals, rather than simply reflecting error of measurement.

Laterality and language are multidimensional constructs, and in future work FTCD could be used to test bilingual laterality with different tasks and larger, more heterogeneous samples, differing on what DeLuca *et al.* (2019) referred to as "the spectrum of experiences". As an inexpensive, non-invasive, comfortable, easily applicable, mobile, and child-friendly method, with a high temporal resolution, FTCD can complement fMRI, allowing us to test large samples and track changes throughout development, with repeated administration and with different tasks.

## Data availability

### Underlying data

Open Science Framework: Bilingual FTCD, https://doi.org/10.17605/OSF.IO/VD9DT ( Bishop *et al.*, 2021a).

This project includes the original raw and processed data for study 1. Please see the Data Dictionary for a description of the files.

Open Science Framework: Lateralisation in bilinguals, https://doi.org/10.17605/OSF.IO/MDCZ5 ( Bishop *et al.*, 2021b).

This project includes the raw data for study 2 and processed data for studies 1 and 2, with custom scripts used for analysis.

Data are available under the terms of the Creative Commons Zero "No rights reserved" data waiver (CC0 1.0 Public domain dedication).
