# Supplementary material for: Cerebral lateralisation of first and second languages in bilinguals assessed using functional transcranial Doppler ultrasound
Source: Wellcome Open Res. 2021 Jul 28;1:15. Originally published 2016 Nov 15. [Version 2] doi: 10.12688/wellcomeopenres.9869.2 (PMC8361806; doi:10.12688/wellcomeopenres.9869.2)
Supplement: Peer review history

# Peer review: Reviewer response for version 2

**DOI:** 10.21956/wellcomeopenres.18790.r45183

**Published:** 2021-08-12

**Authors:** Marc Brysbaert

**Affiliations:** 1 Department of Experimental psychology , Ghent University, Ghent, Belgium

This is an article I was critical about, because I did not think the question was worthwhile nor that the method was adding much information. At the same time I indicated I did not mind the data being published, as they are sound and can be useful in a review article (if only to remind researchers of the fact that the hypothesis of different lateralisation of L2 is wrong). The authors have responded well to the comments made by the reviewers and added extra data. Therefore, I am happy to approve for indexing.

Reviewer Expertise:

NA

I confirm that I have read this submission and believe that I have an appropriate level of expertise to confirm that it is of an acceptable scientific standard.

# Peer review: Reviewer response for version 1

**DOI:** 10.21956/wellcomeopenres.10640.r20237

**Published:** 2017-03-20

**Authors:** Andreas Jansen, Verena Schuster

**Affiliations:** 1 Laboratory for Multimodal Neuroimaging (LMN), Department of Psychiatry, University of Marburg, Marburg, Germany; 2 Department of Psychiatry, University of Marburg, Marburg, Germany

Although functional asymmetries between the hemispheres have been known since the mid-19 ^th^ century, we still lack a thorough understanding of the underlying mechanisms. In particular, we do not have precise models that reveal which factors drive hemispheric specialization, how lateralization processes of different cognitive functions interact with each other, and how the brain integrates processes that are lateralized to opposite hemispheres. In the present study, Grabitz and colleagues aimed to investigate whether the hemispheric lateralization of first and second languages is different. Hemispheric dominance was assessed by functional transcranial Doppler sonography (fTCD) in 26 high proficiency bilinguals with either German or French as their first language (L1) and English as their second language (L2). fTCD was used to assess task-dependent blood flow velocity changes in the left and right middle cerebral arteries during a cued word generation task. The authors report that the majority of participants (22/26) were significantly left lateralized for both L1 and L2. They found no significant difference between the lateralization of L1 and L2, as assessed by a lateralization index (LI). They conclude that in highly proficient bilinguals, there is strong concordance for cerebral lateralization of first and second languages. Although the study was competently performed, there are some concerns about the conceptual planning of the study and the application of fTCD.

Conceptual foundations of the study: There are many aspects of functional neuroanatomy that might differ between L1 and L2, for instance the recruitment of brain regions, the strength of brain activity in specific regions or the connectivity between language regions. Hemispheric lateralization is only one aspect. It might have been useful to explain why the authors assessed in particular hemispheric dominance, it might have been useful to state why they anticipated that language lateralization is stronger for a bilingual person’s first language than for the second language, and it might be have been useful to explain what they authors would have had concluded when they had found significant differences between the lateralization of L1 and L2 – expect that there are significant differences. To interpret non-significant differences, as in the present study, it is also necessary to explicitly state how strong the LI would be expected to differ between L1 and L2. What is a minimal difference that would have been considered as relevant? It is also not clear whether the authors intended to assess differences between L1 and L2 on a group level or in individual subjects? What would be the putative role of interindividual differences? In summary, in its present version of the manuscript a theoretical concept is completely missing. Without this concept, it is not possible to properly interpret the findings. The manuscript gives the impression that the authors were just looking for differences in a rather exploratory way.

Application of fTCD: Before performing a study, it might be a useful exercise to ask whether the imaging technique used is a suitable tool to answer the question asked. I have serious doubts that fTCD can be applied for that purpose. The authors expect to find differences between the lateralization of L1 and L2. It is important to know whether the technique is sufficiently sensitive to find differences, if they exist. As mentioned before, the authors do not explicitly state what differences they expect. In our opinion, it is rather unlikely that the hemispheric dominance (left, right, bilateral) of L1 and L2 will be different. If a subject is for instance left dominant for L1, we do not expect that she will be right dominant for L2. The expected differences will most likely be on a smaller scale. A subject that is left dominant for L1 might be a bit less left dominant for L2. Is fTCD able to find these differences? Unfortunately, there are no methodological studies that assessed how sensitive fTCD is to find potentially small differences in the degree of lateralization. We certainly agree that fTCD is a useful tool to determine hemispheric dominance (that is, left- or right-hemispheric lateralization). It is, however, unknown if the technique can be used to also assess small differences in the degree of hemispheric lateralization. Large methodological studies in this regard, in particular from independent groups (i.e., not from the developers of AVERAGE), are missing. One might also ask why the developers report correlations between fTCD and other techniques (such as fMRI or the Wada test) as high as r~0.9 (and even much higher), when it is not possible to reproduce these findings even with the same modality. Furthermore, fTCD assesses blood flow velocity changes in the vascular territory of the left and right middle cerebral artery. This territory however shows a high interindividual variability. While one might argue that main network nodes of the language system, such as “Broca’s area”, lie within this territory in all subjects, other regions that are also active during the task might be included in the calculation of the LI in some subjects, but not in others. What are the consequences when one compares a LI between subjects? In summary, it is unclear whether fTCD is sensitive enough to measure small differences between the lateralization of L1 and L2.

 To conclude, the study deals with an interesting topic and is competently performed. However, the theoretical foundation should be described in more detail, the expected difference between the LI of L1 and L2 should be reported, and it should be made clear that fTCD is able to measure the expected differences at all.

Reviewer Expertise:

NA

We confirm that we have read this submission and believe that we have an appropriate level of expertise to state that we do not consider it to be of an acceptable scientific standard, for reasons outlined above.

# Response

**Published:** 2021-06-24

**Authors:** Dorothy Bishop

**Affiliations:** University of Oxford, UK

*‘explain why the authors assessed in particular hemispheric dominance’ – many reasons for differences in L1 and L2: recruitment of brain regions, strength of brain activity, connectivity between language regions*

 We now make it clear that we recognise that there are potentially many ways in which language processing may differ for the two languages in bilinguals, but we do not think that invalidates a decision to look specifically at brain lateralisation, which has previously been discussed as potentially differing between languages.

*State why it was anticipated that language lateralization is stronger for a bilingual persons L1 than for L2..   Explain what the authors would have concluded when they had found significant differences between the lateralisation of L1 and L2. Explicitly state how strong the LI would expected to differ between L1 and L2.*

 We now go into more detail regarding predictions from prior literature. The prediction of discrepant laterality between languages was not strong: In the literature, there are reports of both the same strength of lateralisation for L1 and L2 and also reduced lateralisation for L2.  A finding of significant difference in lateralisation between L1 and L2 would have lent further support to one side of this debate. 

*What is a minimal difference that would have been considered as relevant? There are issues with interpreting non-significant findings.*

 As well as reporting Bayes Factors for mean comparisons, we have now conducted further analysis using the Bland-Altman method, which is specifically designed to address this issue.

*It is also not clear whether the authors intended to assess differences between L1 and L2 on a group level or in individual subjects? What would be the putative role of interindividual differences?*

 This is a within-subjects study, with each person tested in both their languages, so the differences are evaluated in individual subjects. The correlations that are reported depend on there being individual differences in the extent of lateralisation. The result, therefore, hinges on interindividual differences.

*Important to know if technique is sensitive to find differences if they exist – are there methodological studies to assess the sensitivity of fTCD to small differences in lateralisation?  ' I have serious doubts that fTCD can be applied for that purpose. The authors expect to find differences between the lateralization of L1 and L2. It is important to know whether the technique is sufficiently sensitive to find differences, if they exist.'*

 Since this study was conducted, we have reported a study of test-retest reliability of laterality indices assessed using fTCD, which we now cite.  They are high enough to give confidence that the degree, as well as direction of laterality measured this way, is reasonably stable. See Woodhead, Z. V. J., Bradshaw, A. R., Wilson, A. C., Thompson, P. A., & Bishop, D. V. M. (2019). Testing the unitary theory of language lateralization using functional transcranial Doppler sonography in adults. *Royal Society Open Science*, *6*(3), 181801. https://doi.org/10.1098/rsos.181801.

 The reviewers clearly have a very negative impression of fTCD as a measure of laterality, but it's unclear what they prefer. The Wada technique is a blunt instrument that is useful in clinical contexts for making a basic distinction between left, right and bilateral, but it is neither feasible nor useful for measuring degrees of lateralisation. With fMRI one can quantify the LI, but the results will depend on the statistical approach (e.g. height or extent of statistic, %signal change etc), ROI studied and on thresholding. The kinds of individual difference in vasculature that the reviewers mentioned may well affect the observed LI - we now make that point in the Discussion. However, this will be as true for measures from fMRI as for fTCD, and in addition, with fMRI, the issue is complicated by the possibility of individual differences in localisation of language regions. 

 So, while we accept that fTCD is not perfect, neither are other methods, and part of our goal in ongoing research is to use them as complementary methods. Indeed we regard it as a worthwhile endeavour in future to consider how far the LI in fTCD relates to anatomical variation. But we don't see any of these as reasons to dispense with the results we have obtained, which we regard as part of a complex pattern of evidence on these issues.

*Why did the developers report correlations between fTCD and other techniques (such as fMRI or Wada test) as high as r~0.9, when it is not possible to reproduce these findings even with the same modality?*

 We cannot say why the Münster group who developed fTCD reported these correlations.  Our work is independent of theirs and we have not used the Average software for some years, though the processing steps we adopt are largely the same. The correlations they originally reported were based on small sample sizes and would have large confidence intervals around them. In addition, language laterality, as conventionally measured,  is usually not normally distributed and should be evaluated with a nonparametric correlation coefficient. We hope to obtain data on larger samples in future that will provide more solid evidence on the relationship between lateralisation as assessed by fTCD and fMRI.

# Peer review: Reviewer response for version 1

**DOI:** 10.21956/wellcomeopenres.10640.r18023

**Published:** 2016-12-05

**Authors:** David W. Green, Tom Hope

**Affiliations:** 1 Experimental Psychology, Faculty of Brain Sciences, University College London, London, UK; 2 Wellcome Trust Centre for Neuroimaging Institute of Neurology, University College London, London, UK

This is a succinctly written paper reporting the novel results from a non-invasive technique (functional transcranial doppler ultrasound) that examines changes in blood flow velocities in the left and right middle arteries in response to a cued word production task in a person’s native language (L1, either French or German) and in their second language (L2, English). The participants were young proficient bilingual speakers immersed in an English context. The aim was to examine the degree of lateralisation in response to this task in L1 and in L2.  The data are appropriately analysed with suitable correction for the number of comparisons made where required.

**Rationale**

 It is important to deploy non-invasive methods that can be used to assess brain response for a particular tasks in children and in adults. The specific question addressed concerns the extent to which L1 and L2 reveal a comparable pattern of asymmetry as revealed by the measure of blood flow velocity.

 It is worth noting that both hemisphere play a role in speech processing in monolingual speakers. Functional imaging data are consistent with the idea that regional activation during speech production is bilateral for motor, premotor, subcortical, and superior temporal regions whereas middle frontal activation is predominantly left lateralised (Price, 2010). As the authors correctly note, neuroimaging data strongly implicate common regions in the processing of L1 and L2. Indeed from a neurocomputational point of view, there is no reason to envisage that the processing of a second language would recruit radically distinct regions (Green, 2003). Instead, different languages may recruit different microcircuits within common regions (e.g., Paradis, 2004).  We should then expect differences attributable to the distinct phonological and syntactic properties of words in different languages and commonalities in terms of their reference to common entities. Consistent with this possibility, Correia *et al.* (2014), using multi-voxel pattern analysis, reported discriminating neural response in multiple temporal, parietal and frontal cortical regions to individual spoken animal nouns (horse/duck) in English and Dutch combined with an invariant response pattern to the translation equivalents (paard/eend) indicative of access to common semantic/conceptual knowledge in regions such as the anterior temporal pole. In modelling recovery post-stroke, we found that models implicating the same brain regions were equally predictive for both monolingual and bilingual speakers displaying parallel recovery patterns (Hope *et al.,* 2015). Evidence for selective recovery post-stroke does not contradict this position, but rather points to a difficulty in control (Green, 2008). Detailed determination of this possibility in the context of speech production awaits future research. However, the Wada test (using injection of intracarotid amobarbital), referred to by the authors as the gold standard in determining lateralization, strongly implicates left hemisphere representation for both languages of a bilingual speaker (e.g., Rapport, Tan & Whitaker, 1983).  A non-invasive method as reported here provides a useful adjunct despite its noted limitations in terms of identifying the microcircuits involved.

**Participant information**

 Self-reported proficiency does generally correlate reasonably well with more objective measures as the authors note. Nevertheless, it is usually desirable to report such objective measures. For instance, for vocabulary measures the various tests under the rubric of LexTale offers a good source (Brysbaert, 2013; Lemhöfer & Broersma, 2012). There are also Quick Placement tests to assess syntactic knowledge.

**Procedure**

 Given the experimenter spoke English all the time how was the transition to the word generation task managed, in particular the switch from describing a picture in L1 to the naming task?

**Data analysis**

 The word generation task involved an interval for the silent generation of words in response to a cued letter (15 secs) followed by a 5 second recall interval. Although this interval is short and so constrains information on relative difficulty, it is of interest to know the mean scores and their variance. If there is variance, does such variance have detectable effects on the signal?

**Estimates of reliability**

 The authors nicely use odd-even trials to estimate signal reliability for the asymmetry index. This estimate proved significant for the production task in the native language (L1) but not for the second language, English (L2). If there is no asymmetry difference then shouldn’t there be a significant correlation when alternate trials are taken from different language runs?

Reviewer Expertise:

NA

We confirm that we have read this submission and believe that we have an appropriate level of expertise to confirm that it is of an acceptable scientific standard.

# Response

**Published:** 2021-06-24

**Authors:** Dorothy Bishop

**Affiliations:** University of Oxford, UK

*Comments under Rationale*

 We thank the reviewers for providing an overview of the literature on microcircuitry, which we now mention in the Discussion

*Usually desirable to report objective measures of language proficiency*

 Please see response to reviewer 1. We did not have such measures for Study 1, but we do for study 2.

*Given the experimenter spoke English all the time, how was the transition to the word generation task managed, in particular the switch from describing a picture in L1 to the naming task?’*

 Alas, this was not noted at the time of data collection for Study 1 and we do not have a record of how this was handled, though we do state that the examiner used English throughout. For Study 2, the experimenter switched instruction languages according to the tested languages.

***Data analysis. **The word generation task involved an interval for the silent generation of words in response to a cued letter (15 secs) followed by a 5 second recall interval. Although this interval is short and so constrains information on relative difficulty, it is of interest to know the mean scores and their variance. If there is variance, does such variance have detectable effects on the signal?*

 We were not sure we had interpreted this correctly; in Study 1 words were generated covertly, therefore we did not have a record of responses. However, in previous studies with monolinguals, we have specifically considered whether varying task difficulty affects laterality. Where difficulty is varied by constraining the task (requiring words starting with 2 specific letters rather than one), this reduced performance but did not affect the LI. (Badcock, N. A., Nye, A., & Bishop, D. V. M. (2012). Using functional transcranial Doppler ultrasonography to assess language lateralisation: Influence of task and difficulty level. Laterality, 17(6), 694–710. https://doi.org/10.1080/1357650X.2011.615128). In Study 2 subjects generated words overtly and we report data on number of words produced.  There was no relationship between number of words generated and LI. 

***Estimates of reliability **The authors nicely use odd-even trials to estimate signal reliability for the asymmetry index. This estimate proved significant for the production task in the native language (L1) but not for the second language, English (L2). If there is no asymmetry difference then shouldn’t there be a significant correlation when alternate trials are taken from different language runs?*

 We were also puzzled by the differing estimates of split half reliability - as it turns out when we reanalysed the data for this version, using our current analysis scripts, the estimate of split half reliability was more similar for the two languages: for L2, the original analysis gave r = .28. With our new method, one participant met criteria as an outlier and was excluded, and we also used Spearman rather than Pearson correlation, and based the LI on the mean rather than peak of the difference waveform; this gives r = .60. Please note: the analytic decisions leading to these changes were made a priori: we used the scripts and outlier exclusion criteria that we documented in Woodhead et al (2019), and list here how each modification of the method affected the correlation:

 - Discarding one participant with noisy data (participant 14), R = 0.44

 - Using Spearman’s correlations instead of Pearson’s, R=0.49

 - Using mean LI method instead of peak, R=0.60

 We feel this provides further justification for basing analyses on mean rather than peak values: the latter can be more noisy, especially if the data do not show a single pronounced peak.

# Peer review: Reviewer response for version 1

**DOI:** 10.21956/wellcomeopenres.10640.r17649

**Published:** 2016-12-05

**Authors:** Marc Brysbaert

**Affiliations:** 1 Department of Experimental psychology , Ghent University, Ghent, Belgium

It pains me to have to write this review. The research done is good and reliable (and hence the Wellcome Trust may decide to publish it), but the question addressed is futile and the methods used far from optimal. Therefore, I fear that if this article is indexed to PubMed Central, it will not do the authors much good.

 For a start, the authors had anticipated that language lateralization might be stronger for a bilingual's first language than for the second language. In 1992, Paradis already called this the Loch Ness Monster of research on laterality and bilingualism. There is no sound evidence whatsoever that L2 processing would be less lateralized than L1 processing (as the authors indeed found). There is even very good evidence that as L2 proficiency increases, it increasingly uses the very same brain areas as L1 processing. Only at low levels of L2 proficiency can one sometimes see extra right and left hemisphere activity, arguably because the participants are using all types of strategies, including non-language ones.

 Second, the authors are using the crudest neuroscientific technique available, fTCD. As they say, it is cheap, it can be applied easily (but leads to a considerable loss of participants), but it is also very crude, as it only compares to blood flow to the left vs. the right hemisphere. In the present study, the reliability is good (except for L2 processing), but even so it remains a technique that only can tell you something about more left than right processing, nothing more. So, in the end the authors are investigating a strawman hypothesis with an unformative technique.

 Third, there are power issues. A lot of subject-related variables are tested on a group of 26 participants. Luckily the authors did not find anything significant, because any significance they would have found, would have been very likely due to a statistical fluke, which cannot be replicated (see papers by Gelman). 

 Fourth, individual differences are thought to be of interest. Still, they are studied with subjective scales. Why not measure the proficiency with a vocabulary test (e.g., LexTALE, Shipley)? Why use Likert scales? In several studies (involving the French and Spanish Lextale tests), I've reported that although there is a good correlation between subjective estimates on the basis of a Likert scale and the Lextale scores, for individual participants there can be a big difference, because participants use different comparison groups (e.g., L2 learners compare their performance to other L2 learners, not to L1 speakers). If the authors want to keep on using subjective measures, they may want to try descriptions as the levels defined by the European framework.

 As said, if the goal of the Wellcome Open Research initiative is to make all reliable empirical data available, I am not against publication. However, for the above reasons I do not think this publication will do the authors (nor the journal) much good. The only bit of information I found of value was Figure 4. Even then it would be good to see this supported by fMRI validation. I know the Knecht group did so, but still I'd like to see it done in other groups as well. I'm very curious, for instance, to what extent the bilateral patterns are valid. We rarely see them in fMRI research.

Reviewer Expertise:

NA

I confirm that I have read this submission and believe that I have an appropriate level of expertise to confirm that it is of an acceptable scientific standard, however I have significant reservations, as outlined above.

# Response

**Published:** 2021-06-24

**Authors:** Dorothy Bishop

**Affiliations:** University of Oxford, UK

Brysbaert, while unenthusiastic about our original paper, accepted that "if the goal of the Wellcome Open Research initiative is to make all reliable empirical data available, I am not against publication." Nevertheless, he queried whether the study was worth doing, given that prior work with fMRI had not found discordant laterality for two languages in bilinguals. In addition, it was felt that reliance on self-reported proficiency was non-optimal, and that there were concerns about concluding a lack of difference between languages when sample size, and hence statistical power, was low. He also expressed misgivings about the method we had used, functional transcranial Doppler ultrasound.

*1. Prediction of different laterality in L1 and L2 is futile. We already know that is not the case.*

 If we understood this point correctly, the reviewer is arguing that the fact that laterality is the same in L1 and L2 for proficient bilinguals is so well established that it is pointless to provide a further demonstration of the point. We disagree. The literature has not always been consistent and most studies are small, so an accurate picture may only become clear when there is sufficient information for a meta-analysis. Our aim was to use fTCD to contribute to this literature.  We accept that the reviewer has a very low opinion of fTCD, but we do not think this is justified, and indeed would argue that the strong correlations between L1 and L2 laterality indices obtained with this method provide some evidence that individual variation in degree of lateralisation are meaningful. 

*2. fTCD is not sensitive enough to detect relevant hemispheric differences*

 We now provide more arguments in support of fTCD. We note also reviewer 2's comment: ' A non-invasive method as reported here provides a useful adjunct despite its noted limitations in terms of identifying the microcircuits involved.'  Brysbaert states that fTCD only tells you about left- vs right hemisphere blood flow. That's exactly what we are interested in, so this criticism does not seem valid. We should stress we are not making massive claims for fTCD - it clearly has its limitations -  but dismissing a study on laterality just because it uses this method seems premature.  It is one tool in the range of possible methods: we need to do more work with all of them (behavioural, fTCD, fMRI) to study how they relate to one another and how reliable and sensitive they are, in order to make progress in laterality research.  This is exactly what we are doing in our current research programme. 

*3. ‘there are power issues. A lot of subject-related variables are tested on a group of 26 participants.’*

 We agree that there are power issues. Brysbaert claims the result is unlikely to replicate. We have now included Study 2 - this confirms that the result does replicate, and generalises to another language and task (semantic fluency). 

 In terms of the subsequent exploratory analysis of correlations with other measures, as noted by reviewer 2: ' The data are appropriately analysed **with suitable correction for the number of comparisons made where required.**' (our emphasis). However, we agree the sample is too small of sensible exploratory analyses, and have now modified our focus to the Age of Acquisition effect, which is a matter of some debate in the literature.  Other variables are reported for completeness, but we agree that it is not sensible to report all correlations in the absence of a priori predictions.

*4. ‘individual differences are thought to be of interest. Still, they are studied with subjective scales. Why not measure the proficiency with a vocabulary test (e.g., LexTALE, Shipley)? Why use Likert scales? In several studies (involving the French and Spanish Lextale tests), I've reported that although there is a good correlation between subjective estimates on the basis of a Likert scale and the Lextale scores, for individual participants there can be a big difference, because participants use different comparison groups (e.g., L2 learners compare their performance to other L2 learners, not to L1 speakers). If the authors want to keep on using subjective measures, they may want to try descriptions as the levels defined by the European framework’*

 We agree. This point was also made by Reviewers 2, though they also pointed out that self-reported proficiency correlates reasonably well with more objective measures. A strength of study 2 is that it used more objective measures of language proficiency.  We thank the reviewer for the excellent suggestions for measures to be used in future work.
